# Deep behavioral phenotyping tracks functional recovery following tibia fracture in mice

**DOI:** 10.3389/fphys.2025.1630155

**Published:** 2025-08-26

**Authors:** Jonathan E. Layne, Dustin M. Snapper, Molly E. Czachor, Charles Lam, Jacob D. Matityahu, Dane R. G. Lind, Matthieu Huard, Johnny Huard, Kazuhito Morioka, Julian C. Motzkin, Allan I. Basbaum, Jarret A. P. Weinrich, Chelsea S. Bahney

**Affiliations:** ^1^ Department of Orthopaedic Surgery, UCSF Orthopaedic Trauma Institute, San Francisco, CA, United States; ^2^ Center for Regenerative Medicine, Steadman Philippon Research Institute, Vail, CO, United States; ^3^ UCSF Department of Neurology and Weill Institute for Neuroscience, San Francisco, CA, United States; ^4^ UCSF Department of Anesthesia and Perioperative Care, San Francisco, CA, United States; ^5^ UCSF Department of Anatomy, San Francisco, CA, United States

**Keywords:** behavioral phenotyping, fracture repair, fracture-related pain, machine-learning image analysis, sexual dimorphism

## Abstract

**Introduction:**

An estimated 178 million fractures occur worldwide annually, with lower limb fractures showing high rates of poor healing, often resulting in reduced mobility and chronic pain. Bone healing and the ability to bear weight are closely tied to the mechanical stability of the fracture site. Although fracture stabilization is a well-established factor modulating bone repair, there remains a notable gap in sophisticated non-destructive technologies that can rapidly and objectively quantify functional recovery in preclinical settings. We introduce a novel behavioral phenotyping approach enabling rapid quantification of post-fracture weightbearing and kinematic metrics in freely behaving mice. Our goals were to identify and characterize metrics most indicative of fracture-induced behavioral impairment and to use these metrics to quantify how functional recovery is altered in mice with pin stabilized *versus* non-stabilized fractures. We also explore sex-specific contributions to recovery.

**Methods:**

Male and female C57BL6/J mice received mid-shaft tibial fractures that were either unstabilized or fixed with intramedullary pins; non-fractured mice served as controls. Behavioral recordings were acquired pre-fracture and throughout healing (5–35 days post-fracture). To track mice and analyze changes in paw pressure and kinematics, we performed machine learning-enabled behavioral phenotyping.

**Results:**

Overall, mice with pin-stabilized fractures exhibited less behavioral impairment than mice with unstabilized fractures. Pin stabilization allowed increased weightbearing and produced smaller changes in kinematic metrics. By contrast, we observed only minor sex-specific differences in impairment and recovery following fracture. Our analysis revealed that functional recovery is more complex than individual parameters viewed in isolation, with different parameters identifying distinct recovery timeframes. Therefore, we developed a comprehensive, unified graph theoretic metric encompassing all behavioral parameters. This unified approach confirmed increased severity in unstabilized fractures and identified clear functional recovery windows for both fracture groups.

**Discussion:**

This methodology forms a foundation for future mechanistic experiments focused on biological and mechanical variables influencing functional healing and enables more rapid testing of strategies to accelerate bone healing.

## Introduction

Bone fractures are among the most common orthopedic injuries, with lower limb fractures accounting for 47.3 million fractures globally in 2019 (Collaborators, 2021). Delayed healing, or failure to heal, is especially common in lower limb fractures, with complications reported to occur in 13.6% of femur and 11.7% of tibia fractures. Ekegren et al. (2018) Many non-modifiable factors affect the rate of bone union, such as fracture pattern, degree of soft tissue damage, age, sex, smoking status, and medical comorbidities (Hellwinkel et al., 2020). The orthopaedic surgeon has the greatest control over the mechanical environment of the fracture site, through implant choice, insertion method, and timing of post-operative weightbearing. Mechanical loading is essential for effective bone healing and reduces the risk of delayed union in lower-limb fractures. For this reason, partial and full weightbearing is encouraged as tolerated (Ma et al., 2023). However, increased pain levels can lead to decreased weightbearing and less effective participation in physical therapy, which can negatively impact recovery (Eickhoff et al., 2022; Majuta et al., 2015).

Long bone fractures heal through four distinct but overlapping biological phases (Marcucio et al., 2023; Molitoris et al., 2024a). Briefly, following fracture, a hematoma forms, which stops bleeding, contains bone fragments, and triggers a pro-inflammatory cascade critical to the repair response (Kolar et al., 2010; Xing et al., 2010). In mice, this pro-inflammatory phase typically spans the first 5 days post fracture. Bone healing then proceeds through two distinct processes: (1) along the cortical surfaces of the bone, skeletal progenitor cells differentiate to form bone directly, whereas (2) within the fracture gap, progenitors differentiate to chondrocytes and form a provisional cartilage matrix that bridges the fracture (Colnot, 2009). Cartilage in the fracture gap then transforms to bone, through the process of endochondral ossification (Bahney et al., 2014; Haffner-Luntzer et al., 2019; Hu et al., 2017; Wong et al., 2020a; Wong et al., 2020b; Julien et al., 2020; Zhou et al., 2014). In the final phase of healing, osteoclasts remodel the newly formed trabecular bone into cortical bone (Drissi and Sanjay, 2016).

Physical forces are significant contributors to healing progression (Morgan et al., 2008; Anani and Castillo, 2022; Augat et al., 2021). Early in fracture healing, local mechanical strain—shaped by the fracture pattern and fixation method—directs cellular proliferation and lineage differentiation (osteogenic, chondrogenic, or fibrotic). Later, gross loading of the fracture site, typically in the form of weightbearing, is critical to bone remodeling and consolidation. While moderate compressive forces are favorable for fracture healing, too little or too much compressive loading, torsional and sheer forces, can delay healing. Although the loads and geometry differ between mice and humans, the underlying strain principals are comparable, supporting the use of the mouse as a suitable preclinical model to study functional recovery.

What remains poorly understood is how the biological process of fracture healing correlates with functional behaviors in mice. And, importantly, there is a growing body of evidence suggesting that the paucity of preclinical functional outcome measures in fracture repair hinders translation of effective treatments from mice to humans (Barr and ett, 2015; Woolf, 2010; Clark, 2016; Shen et al., 2020; Gunderson et al., 2020). Standard quantifiable preclinical outcome measures of bone healing are rarely based on behavioral assessment, but rather are destructive, including, histological tissue evaluation, *ex vivo* microcomputed tomography (μCT), gene and protein expression analysis, and biomechanical bone quality testing. These analyses are time consuming, expensive, and typically require a large number of animals to obtain the different destructive data sets. Gait analysis (e.g., DigiGate or Catwalk), static and dynamic weight bearing assays, or scored locomotion scales (e.g., Basso-Beattie-Bresnahan) have been used infrequently in fracture healing studies, due to the specialized equipment needs, the challenge in applying these protocols to mice with fracture, and their time-consuming analysis (Haruki Nishimura et al., 2025; McVeigh et al., 2020). For this reason, there remains an important technology gap for rapid, unbiased, and non-destructive evaluation of clinically informed outcome measurements that can provide more quantitative assessments of fracture-induced pain and functional recovery.

Here, we present data from a longitudinal behavioral phenotyping study in mice in which we quantitatively track functional recovery (i.e., weightbearing and kinematic shifts) after long bone fractures of the lower limb. Our goal was to assess the impact of mechanical stability and sex on functional behavioral recovery after tibia fracture. To represent the clinically modifiable mechanical environment of the fracture site, we compared two different methods of fracture fixation, namely, intramedullary pin stabilized *versus* unstabilized fractures. Additionally, we assessed the contribution of the non-modifiable factor of sex, as presently the preclinical and clinical data are conflicting as to the influence of sex in fracture healing (Haruki Nishimura et al., 2025; Egol et al., 2009; Noori et al., 2020). First, using our behavioral phenotyping approach, we analyzed a large number of individual behavioral metrics. Next, we developed a novel graph theoretic approach that integrates many distinct outcomes into a single, comprehensive metric that quantifies the global behavioral state of each mouse during recovery from fracture. We hypothesize that a unified metric of post-fracture behavior will make it possible to establish the time window for functional recovery and that this metric will confirm sex-independent faster functional recovery in mice with modulated mechanical strain (i.e., stabilized fracture).

## Materials and methods

### Animal husbandry and ethical approval

As recapitulating the physiology of fracture repair and characterizing the behavioral and kinematics of recovery are not feasible using *in vitro* systems or modeling, we used adult (10–14-week-old) male and female wild-type C57BL6/J (Jackson Laboratories Stock #000664) mice or all experiments. All experiments complied with ethical regulations and protocols and were approved by the Institutional Animal Care and Use Committee (IACUC) at our university. All mice were group-housed, provided environmental enrichment, fed a standard diet, and maintained in facilities with standard light/dark cycle and appropriate environmental controls, which ensured the highest standard of care.

### Surgical procedures

Mice either received an unstabilized or intramedullary stabilized tibia fracture. Non-fractured mice were used as a control group. Mice were anesthetized prior to fracture using isoflurane inhalation (4%–5% induction, 2%–3% maintenance). All surgeries were performed on a heated operating table using aseptic technique and ocular ointment was placed on the eyes during anesthesia. Per our approved IACUC protocol, after the surgery, the mice received a single subcutaneous dose of sustained release buprenorphine (3.25 mg/kg, Fidelis Animal Health, Cat#NDC 86084-100-30) for pain control. Following surgery, the mice were socially housed, allowed to ambulate freely and monitored for 72 h for pain and discomfort.

#### Unstabilized tibia fracture

To create a mid-diaphyseal fracture of the right tibia, anesthetized mice were placed pronated under a custom-built three-point bending fracture apparatus. No fixation was provided after the creation of the fractures, which simulate clinical fractures with a high degree of mobility. As previously described, this technique is a well-established method to create robust endochondral repair (Le et al., 2001).

#### Stabilized tibia fracture

Following anesthesia induction, the right leg was shaved and sterilely prepped using three rounds of 70% alcohol wipes, followed by povidone-iodine swab sticks 10%, (Dynarex Corporation, Cat#1202). The knee of the right tibia was placed in flexion and a small skin incision was made superior to the tibial plateau. A 23-gauge needle was used to form a pilot-hole at the top of the tibial plateau. A sterilized insect pin was then inserted through the hole spanning from the tibial plateau through the tibial intramedullary space and secured into the distal tibia. Then, a Dremel was used to create a small hole (0.25–0.5 mm) in the mid-diaphysis of the tibia. To generate a full-thickness tibial fracture, pressure was applied to both the proximal and distal ends of the tibia as previously described (Nelson et al., 2023; Nelson et al., 2024). The pin was then trimmed with wire cutters at the tibial plateau. The incision was closed with 5-0 Biosyn Sutures (Covidien, 5687). Bupivacaine hydrochloride (NovaPlus, RL7562) was applied topically for post-operative pain management.

#### Naïve control mice

Age and sex matched wild-type C57BL6/J mice that received neither anesthesia nor fracture were used as controls for each individual fracture type. These mice were ordered at the same time as the fracture mice, housed identically, and monitored at the same timepoints as their fractured counterparts.

### Behavioral video recording

#### Blackbox device

**FIGURE 1:**
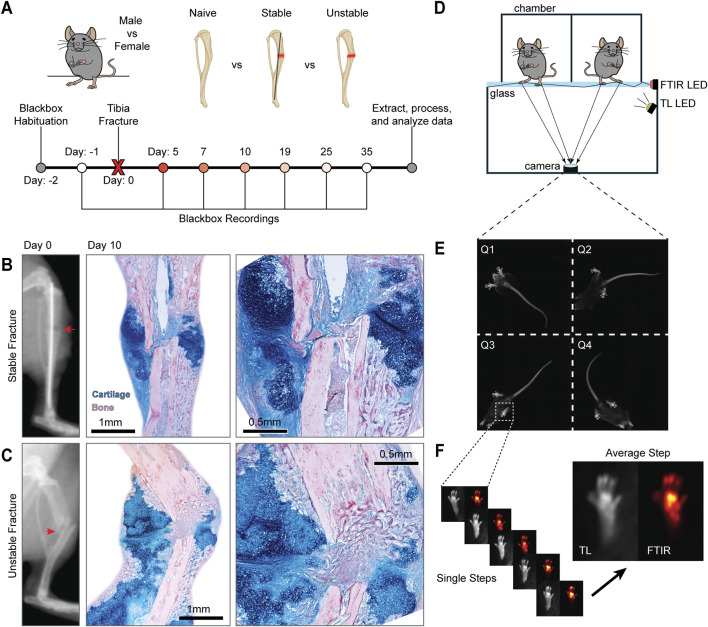
Blackbox monitoring of functional recovery after tibia fracture. **(A)** Schematic representation of experimental groups and Blackbox recording timeline. **(B)** Representative of a pin-stabilized fracture (red arrow) immediately post-operatively (left), followed by representative histology of fracture healing 10 days post-fracture (DPF) processed through Hall-Brundt’s Quadruple (HBQ) stain which indicates cartilage in blue and bone in red. **(C)** Representative radiograph of an unstabilized fracture (red arrow) immediately post-operatively (left), followed by representative histology of fracture healing 10 DPF processed through HBQ stain. The fracture site is indicated in all images with a yellow arrow. **(D)** Diagram of the BlackBox device. The device consists of four chambers that house a single mouse during recording, with two of the four chambers pictured here. The glass floor below the chambers allows for the capture of both transillumination (TL) and frustrated total internal reflectance (FTIR) images. **(E)** Representative frame of the 4-quadrant TL video recordings. **(F)** Representative frames of stepping bouts extracted from the TL and FTIR recordings. Both TL and FTIR images were then averaged across steps to generate a representative image of paw placement pressure distribution during stepping.

We assessed mice for weightbearing and kinematic parameters using the Blackbox R4 device (Blackbox Biotech Inc., BB1R-0015), which captures animal pose and paw pressure. Up to four freely moving mice can be monitored at the same time (Figure 1) (Zhang et al., 2022). Briefly, the Blackbox device encloses a single, high speed, high spatial resolution near-infrared (NIR) camera. The mice are placed onto the glass surface, which holds 4 black acrylic chambers, with only 1 mouse per chamber during recording (Figure 1D). Paw contact force with the glass is transmitted through frustrated total internal reflectance (FTIR). The FTIR light sources are two 850 nm NIR LED strips that are aligned perpendicular to two opposite edges of the glass floor. Transillumination (TL), which enables visual identification of the mouse pose within the chamber, is generated by 4 additional NIR LED strips located 10 cm below the glass floor. The overall frame rate of the camera is set to 90 frames per second (fps). In every other frame, the TL LEDs are turned off, allowing for exclusive imaging of the FTIR signal. This approach produces an effective frame rate of 45 fps (45 TL + 45 FTIR = 90 total fps). The behavioral recording is captured by BlackBox software (Version 0.1.2) onto a Blackbox workstation.

#### Longitudinal behavioral monitoring

Mice were first habituated to the device for 4–5 min for 2 days before fractures were performed and 1 day prior to fracture, we recorded baseline mice behaviors (Figure 1A). Post-fracture behavior recording occurred at 5-, 7-, 10-, 19-, 25-, and 35-day post fracture (DPF). To ensure that the effects of the protocol-required slow-release buprenorphine had worn off, testing began 5-DPF. On the day of testing, animals were placed, one at a time, into an individual Black Box chamber (Figure 1D). Mice were then recorded continuously for 4–5 min. TL and FTIR recordings are saved for further analysis (Figures 1D–F). The duration of individual recording sessions and timing of recordings post fracture were optimized during pilot experiments.

### Automated analysis of behavioral recordings

To analyze data from Blackbox recordings, we developed a custom-written data processing and analysis pipeline within MATLAB (R2023a, MathWorks) that leverages open-source video processing software (FFMPEG) and machine-learning based video object tracking (DeepLabCut [DLC], v1.5.7). The end result of our pipeline is an analysis of weightbearing and kinematics of the mouse. See Table 1 for detailed descriptions of relevant outputs. Data are processed on a Puget Systems Threadripper workstation with an NVIDIA A5000 Ada graphics processing unit (GPU). References to functions below refer to functions native to MATLAB.

**TABLE 1:** Definitions of selected behavioral metrics.

Metric	Description (Please see methods for more details)
Weightbearing Ratio	The ratio of FTIR light intensity of the hindpaws from the fractured over the non-fractured limb
Pad Intensity	The percentage of FTIR intensity restricted to the pad over the entire paw
Stepping Correlation	The Pearson correlation of stepping speeds during walking for indicated paws (i.e., left vs. right hindpaw)
Step Duration	The average duration of a step during walking, defined as the full-width half-max from stepping speed
Maximum Speed	The average maximum speed of the paw during stepping during walking
Step Length	Average length of each step during walking

#### Video processing and automated object labeling

First, it was necessary to split the single Blackbox videos (both TL and FTIR) containing all 4 chambers into individual videos per mouse. This was accomplished using FFMPEG. Split TL videos were then processed through DLC to identify the pose of the mouse within the chamber. The points identified include: hindpaw (both pad and toes), forepaw (pad and toes), base of the tail, abdomen, chest, and mouth (Supplementary Figure S1). Right and left paws are separately identified. To build the pose estimation model, we used videos from 24 behavioral recordings, including videos from both naïve and fracture mice, with at least 40 still frames per video labeled. Using this model, we performed pose estimation (object tracking) on all behavioral videos.

#### Weightbearing analysis

Weightbearing is measured during the stance phase of walking for each paw. A single weightbearing measurement is taken per step, measured 150 milliseconds after the maximum paw speed during stepping. Weightbearing of an individual paw is measured as the summed intensity of paw luminescence within the FTIR video, with paw placement determined from time-synced TL videos, and the FTIR still frame cropped to fit the measured paw. Within a recording session, the summed intensity of weightbearing is averaged across all identified steps. The weightbearing ratio is calculated as the ratio of the mean summed FTIR intensity of compared paws. For example, the hindpaw weightbearing ratio is equal to the ratio of the mean sum of the FTIR intensity of the hindpaw of the fractured limb divided by the hindpaw of the unfractured limb. The weightbearing ratio is calculated for the right vs. left hindpaw, right *versus* left forepaws, right forepaw vs. right hindpaw, left forepaw *versus* left hindpaw, and both forepaws *versus* both hindpaws.

We also calculated the percentage contribution to weightbearing of the toes, pad, and heel within a single paw. Here, the cropped FTIR image used to determine weightbearing is masked to segregate only the contribution to the overall intensity from the particular part of the paw. This masked intensity value is then summed, divided by the overall intensity of the step, and multiplied by 100%, which generates the percent contribution. For the forepaws, only toes and pad contributions are calculated.

#### Kinematic analysis

Next, to extract behavioral endpoints, we analyzed DLC pose for kinematic metrics. We extracted the following paw-related kinematic metrics: maximum paw speed during stepping (cm/s), stride duration (full width half max [FWHM] in milliseconds), and stride length (cm). More general locomotor-related measurements include distance traveled (cm, as measured from the movement of the base of the tail), walking speed (cm/s), and percentage of time spent walking during the recording. The final form of the weightbearing and kinematic metrics is a scalar that represents the average value for each metric within a video (i.e., the average maximum paw speed per paw within a single video). When appropriate, data are normalized to average value of naïve control animals of the same sex (e.g., Figure 4).

#### Graph theoretic analysis

We also developed a unified, comprehensive metric that could integrate changes across all weightbearing and kinematic measurements produced by our analysis. First, for each single scalar metric (for example, walking speed), data from all recordings (both sexes; naïve and both fracture types) are z-scored within-metric. This z-scoring is performed separately for all metrics described above. Next, a matrix of pairwise correlation coefficients (Pearson’s rho and correlation significance values) are computed using the z-scored metrics, comparing each recording to all other recordings in the dataset. The significance matrix is corrected for multiple comparisons using the *MAFDR* function, generating a q-value matrix. A positive adjacency matrix is constructed from the correlation matrix by keeping only pairwise correlations greater than 0.3, and q-values less than 0.05. A weighted graph is then constructed using the *graph* function. Within this generated graph, each node corresponds to a single recording, and edges indicate significant positive correlations between nodes, weighted to account for the strength of the correlation. Finally, we use the *distances* function to calculate the distance (the unit of which is total number of weighted edges that make up the shortest path to connect a given pair of recordings) of the baseline recording to post-baseline recordings within a single animal.

#### Principal component analysis

To perform the principal component analysis, data are first z-scored as described above. Next, a matrix is constructed of individual z-scored metrics to which the *pca* function is applied. The output of this analysis includes PC scores used for plotting and the loading coefficient weights for each metric used to calculate the PC score.

### Fracture radiographs and histology

X-ray radiographs were captured immediately postoperatively with a Faxitron Cabinet X-Ray System (Hewlett Packard, Model#:43855A; 50 kV, 3 mA, 1 min scan time, Figures 1B,C). Fractured tibia were harvested at 10 DPF and fixed in 4% paraformaldehyde at 4 °C. After 24 h, tibias were decalcified in 19% Ethylenediaminetetraacetic Acid (EDTA) and left to rock at 4 °C for 3 weeks with EDTA changes every other day. Decalcified tibias were dehydrated and then embedded in paraffin. Tissue samples were serially sectioned using a Leica RM 2155 microtome at 8–10 μm, with 3 sections per slide. Slides were stained using Hall-Brundt’s Quadruple (HBQ) staining protocol callus (Figures 1B,C) (Hu et al., 2017). Images were captured with a Leica DM5000 B microscope.

### Statistics

Statistical analyses were performed using GraphPad Prism (Version 10.1.2). Data were analyzed for statistical significance using mixed-effect analysis with multiple comparisons and a two-stage linear step-up procedure of Benjamini, Krieger, and Yekutieli. (Benjamini et al., 2006). Data are displayed as the mean ± standard error (SEM). The results of all mixed effects models can be found in the tables provided in Supplementary Material. Symbols are used within the graph to indicate statistical significance with p < 0.05.

## Results

### Unfractured mice exhibit sex-specific differences in weightbearing and kinematics

To understand the presence of sex-specific differences prior to the fracture procedure, we compared gross weights, paw weightbearing, and kinematic parameters of male and female mice at baseline. Age-matched male and female mice exhibit significantly different weightbearing and kinematic profiles (Supplementary Figure S2). Male mice weighed significantly more than females (28.38 ± 2.07 g vs. 22.61 ± 1.539 g, Supplementary Figure S2A) resulting in higher paw luminescence, or the summed FTIR intensity of the whole right hindpaw (Supplementary Figure S2B). This relationship between paw luminescence intensity and mouse gross weight was highly linear (Supplementary Figure S3). Lastly, we found that males have a shorter step duration and step time, and a slower maximum paw speed than female mice (Supplementary Figures S2C–E).

### Stabilization decreases the severity of functional deficits and accelerates recovery

To study functional recovery following tibia fracture, behavioral phenotyping was completed 1-day prior to fracture (baseline), and then 5-, 7-, 10-, 19-, 25-, and 35-day post fracture (DPF, Figure 1). The initial time gap allowed for the effect of anesthesia and slow-release analgesics to wear off. The remaining timepoints followed the mice through the full-time course of healing. Based on the sex-specific differences in weightbearing kinematic parameters at baseline (Supplementary Figure S2), we first analyzed the fracture recovery separately in female and male mice.

**FIGURE 2:**
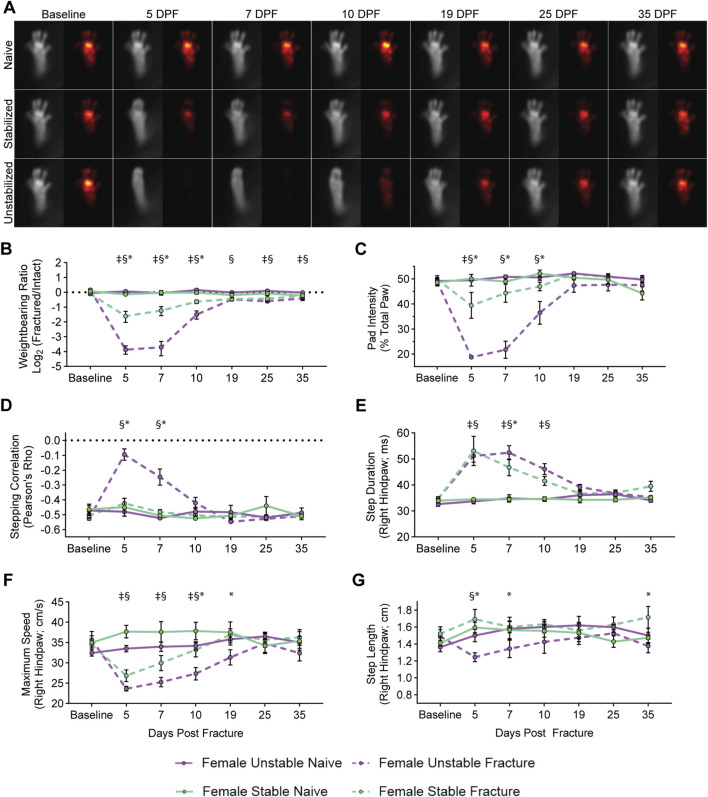
Differential functional impairments in female mice identified by fracture type. **(A)** Average images of paw placement (TL) and weightbearing (FTIR) of naïve, stabilized, and unstabilized fracture females at baseline and 5-, 7-, 10-, 19-, 25-, and 35-day post injury (DPI). Longitudinal analysis of weightbearing and kinematic parameters: **(B)** Weightbearing ratio of the fractured hindlimb to the intact hindlimb (Mixed-effects analysis, Fixed effects: Time (F (6, 48) = 30.89, p < 0.0001) Naïve vs. Fracture (F (1, 8) = 80.68, p < 0.0001). **(C)** Percentage of FTIR intensity localized to the pad of the hindpaw of the fractured hindlimb (Mixed-effects analysis, Fixed effects: Time (F (6, 48) = 25.07, p < 0.0001) Naïve vs. Fracture (F (1, 8) = 24.65, p = 0.0011). **(D)** Hindlimb stepping correlation (Mixed-effects analysis, Fixed effects: Time (F (6, 48) = 14.13, p < 0.0001) Naïve vs. Fracture (F (1, 8) = 7.037, p = 0.0291). **(E)** The average full-width, half-max duration of a single step, in milliseconds (Mixed-effects analysis, Fixed effects: Time (F (6, 48) = 17.39, p < 0.0001) Naïve vs. Fracture (F (1, 8) = 113.2, p < 0.0001). **(F)** Average maximum speed (cm/s) of right hindpaw during stepping (Mixed-effects analysis, Fixed effects: Time (F (6, 48) = 9.228, p < 0.0001) Naïve vs. Fracture (F (1, 8) = 9.027, p = 0.0170). **(G)** Average distance of each step of the right hindlimb in centimeters (Mixed-effects analysis, Fixed effects: Time (F (6, 48) = 0.9560, p = 0.4648) Naïve vs. Fracture (F (1, 8) = 0.0261, p = 0.9134). Significant differences (p < 0.05 after corrections for multiple comparisons) are indicated by: § - unstable fracture and respective naive control; ‡ - stable fracture and respective naive control; * - unstable and stable fracture.

In female mice, we observed that less weight is placed on the paw of the fractured limb for both stabilized and unstabilized fracture groups compared to naïve controls (Figure 2A, Supplementary Videos S1–S3). This decreased weight-bearing on the fractured limb translates to a decreased weight-bearing ratio (ipsilateral fractured limb/contralateral non-fractured limb) for female mice. With both stabilized and unstabilized fracture *versus* naïve controls (Figure 2B). We also recorded further changes in the distribution of weightbearing across the paw of the fractured limb, namely, that the percentage of weightbearing on the pad *versus* the total paw significantly decreases after fracture (Figure 2C). The kinematic assessments similarly uncovered significant changes with fracture. Both fracture types with show increased fractured limb step duration (Figure 2E) and decreased maximum paw speed (Figure 2F). The kinematic analysis further showed that only in mice with unstable fractures are there significant changes in stepping correlation (Figure 2D) and step length (Figure 2G). For all weightbearing and kinematic metrics, the changes induced early in the fracture process recover back to normal over time, albeit at different rates for different metrics (Figures 2A–G).

When comparing female mice with stabilized and unstabilized fractures directly, we observed that unstable fractures produce a more severe functional deficit, and that mice with stabilized fractures recover more quickly. This increased severity of the functional deficit is evident in the degree of change between the weightbearing ratio and pad weightbearing, both of which are less in female mice with stabilized *versus* unstabilized fractures at 5-, 7-, and 10-DPF (Figures 2B,C). Accelerated functional recovery in female mice with stabilized fracture *versus* unstable fracture can also be observed for the pad weight-bearing on the fractured limb. Here the mice with stabilized fracture are no longer significantly different *versus* naïve control mice at 7- and 10-DPF, but are still significantly different from unstable fracture at the same timepoints (Figure 2C). Similarly, in the fractured hindlimb, for both step duration by 5-DPF (Figure 2E) and maximum paw speed by 10-DPF (Figure 2F), we recorded enhanced functional recovery in female mice with stabilized *versus* unstabilized.

**FIGURE 3:**
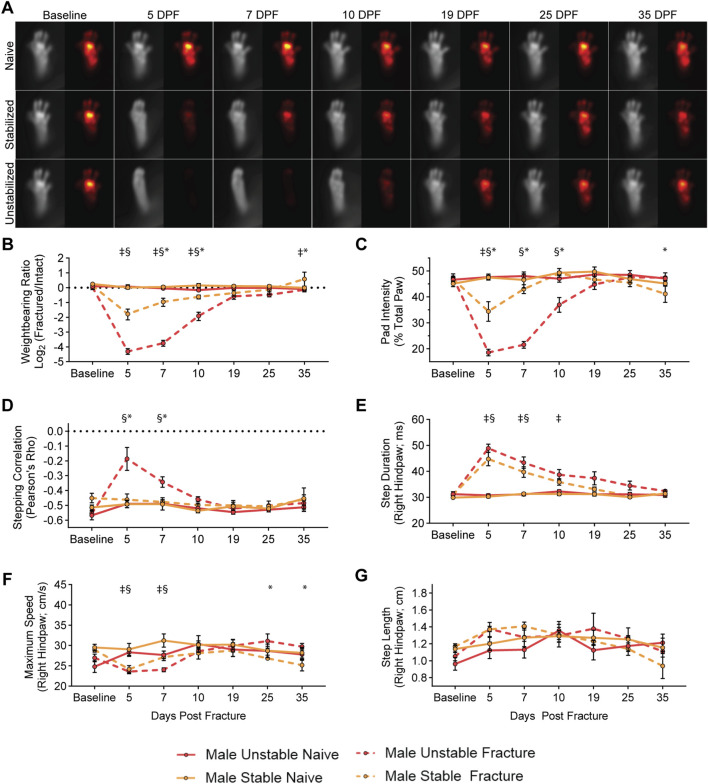
Differential functional impairments in male mice identified by fracture type. **(A)** Average images of paw placement (TL) and weightbearing (FTIR) of naïve, stabilized, and unstabilized fracture males at baseline and 5-, 7-, 10-, 19-, 25-, and 35-day post injury (DPI). Longitudinal analysis of weightbearing and kinematic parameters: **(B)** Weightbearing ratio of the fractured hindlimb to the intact hindlimb (Mixed-effects analysis, Fixed effects: Time (F (6, 42) = 10.79, p < 0.0001) Naïve vs. Fracture (F (1, 7) = 42.48, p = 0.0003). **(C)** Percentage of FTIR intensity localized to the pad of the hindpaw of the fractured hindlimb (Mixed-effects analysis, Fixed effects: Time (F (6, 42) = 24.90, p < 0.0001) Naïve vs. Fracture (F (1, 7) = 64.59, p < 0.0001). **(D)** Hindlimb stepping correlation (Mixed-effects analysis, Fixed effects: Time (F (6, 42) = 6.387, p < 0.0001) Naïve vs. Fracture (F (1, 7) = 11.30, p = 0.0121). **(E)** The average full-width, half-max duration of a single step, in milliseconds (Mixed-effects analysis, Fixed effects: Time (F (6, 42) = 33.92, p < 0.0001) Naïve vs. Fracture (F (1, 7) = 45.07, p = 0.0059). **(F)** Average maximum speed (cm/s) of right hindpaw during stepping (Mixed-effects analysis, Fixed effects: Time (F (6, 42) = 5.237, p = 0.0004) Naïve vs. Fracture (F (1, 7) = 2.898, p = 0.1325). **(G)** Average distance of each step of the right hindlimb in centimeters (Mixed-effects analysis, Fixed effects: Time (F (6, 42) = 5.508, p = 0.0003) Naïve vs. Fracture (F (1, 7) = 0.4581, p = 0.5203). Significant differences (p < 0.05 after corrections for multiple comparisons) are indicated by: § - unstable fracture and respective naive control; ‡ - stable fracture and respective naive control; * - unstable and stable fracture.

In male mice, we observed largely similar changes in weightbearing and kinematics after fracture. In males, we found that unstabilized fracture produces larger changes in weightbearing ratio and pad intensity than stabilized fracture at early fracture timepoints (Figures 3B,C). As in female mice, male mice with unstable fracture demonstrated significant changes in stepping correlation, with no changes observed after stabilized fracture (Figure 3D). Again, at early fracture timepoints, we observed significant increases in step duration and decreases in maximum paw speed (Figures 3E,F). For the kinematic parameters, one notable sex difference was that males lacked distinct changes in the step length, across fracture types (Figure 3G). In terms of the severity of the functional deficit and speed of functional recovery, males follow the similar pattern as the females above (Figures 3A–G).

### Male and female mice exhibit largely similar functional changes after fracture

**FIGURE 4:**
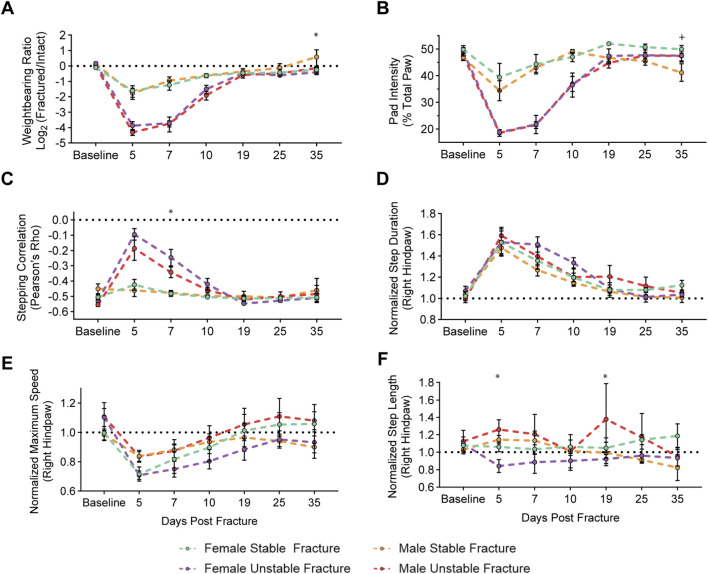
Male and female mice exhibit similar functional impairments and recovery within a fracture type. Longitudinal analysis of weightbearing and kinematic parameters comparing male and female mice after stable and unstable fracture: **(A)** Weightbearing ratio of the fractured hindlimb to the intact hindlimb (Mixed-effects analysis, Fixed effects: Time (F (6, 48) = 10.79, p < 0.0001) Stable vs. Unstable (F (1, 8) = 11.92, p = 0.2580). **(B)** Percentage of FTIR intensity localized to the pad of the hindpaw of the fractured hindlimb (Mixed-effects analysis, Fixed effects: Time (F (6, 48) = 60.35, p < 0.0001) Stable vs. Unstable (F (1, 8) = 16.65, p = 0.0035). **(C)** Hindlimb stepping correlation (Mixed-effects analysis, Fixed effects: Time (F (6, 48) = 26.38, p < 0.0001) Stable vs. Unstable (F (1, 8) = 14.9, p = 0.0055). **(D)** The normalized average full-width, half-max duration of a single step, in milliseconds (Mixed-effects analysis, Fixed effects: Time (F (6, 48) = 44.44, p < 0.0001) Stable vs. Unstable (F (1, 8) = 1.900, p = 0.2054). **(E)** Normalized average maximum speed (cm/s) of right hindpaw during stepping (Mixed-effects analysis, Fixed effects: Time (F (6, 48) = 15.30, p < 0.0001) Stable vs. Unstable (F (1, 8) = 0.3853, p = 0.8493). **(F)** Normalized average distance of each step of the right hindlimb in centimeters (Mixed-effects analysis, Fixed effects: Time (F (6, 48) = 0.7299, p < 0.6278) Stable vs. Unstable (F (1, 8) = 8.901e^−5^, p = 0.9927). Data in **(D–F)** are all normalized to respective naïve control mice. Significant differences (p < 0.05 after corrections for multiple comparisons) are indicated by: + – male and females with stable fracture; * - males and females with unstable fracture.

Next, we sought to directly compare functional recovery patterns between female and male mice following fracture. Within weightbearing metrics, we observed no significant sex-specific differences of the weightbearing ratio or pad weight-bearing distribution during the main recovery period (5-25 DPF) (Figures 4A,B). A minor, yet significant, difference in stepping correlation was observed at 7 DPF identified between female and male mice with unstable fracture (Figure 4C). To account for observed differences in kinematic endpoints identified between the sexes of naïve animals (Supplementary Figure S2) and allow for direct comparisons not influenced by animal size and weight, we normalized kinematic endpoints (e.g., step length, step duration, maximum paw speed) in fractured mice to their respective naïve control groups for each sex and fracture type. While there were no sex differences in the step duration or maximum speed (Figures 4D,E), normalized step length was decreased in females and increased in males 5- and 19-DPF (Figure 4F).

### Graph theory-based behavioral phenotyping identifies post-fracture recovery window

As individual behavioral metrics identify different time windows for functional recovery (for example, weightbearing ratio vs. pad intensity), we developed a unified metric that integrates changes across all measurements calculated with our analysis. This approach provides an unbiased, global assessment of functional recovery during fracture healing. First, to visualize how patterns of behaviors change across our entire dataset, we generated a z-scored heatmap. Hierarchical clustering by behavioral metrics (x-axis) and fracture type, sex, and timepoint (y-axis) identified distinct groupings within our dataset, by fracture fixation and sex (Supplementary Figure S4). Similarly, using a pairwise correlation analysis to compare correlations across all the behavioral metrics between timepoints, we observed 3 distinct groupings within the data. These 3 groups are defined by male sex, female sex, and mice with fracture (Supplementary Figure S5), which is further confirmed by principal component analysis (Supplementary Figure S6).

**FIGURE 5:**
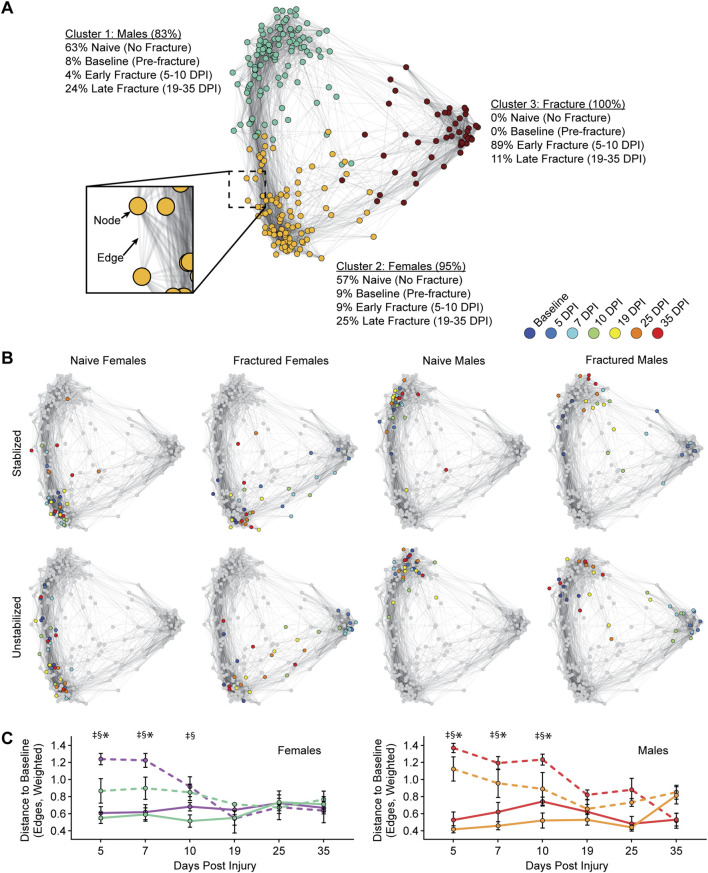
Graph theory-based behavioral phenotyping identifies post-fracture recovery window. **(A)** Functional changes after tibia fracture are plotted using a graph theoretic approach. As represented in the cutout, individual recordings are represented as *nodes* in the graph, and positive correlations between sets of behaviors are represented as *edges*. k-means clustering identifies 3 clusters within the graph (nodes colored by cluster identity), with identities of nodes within each cluster indicated as percentages. **(B)** Representation of node identities on the graph, as indicated by sex and fracture conditions. Colored nodes indicated data from Baseline to 35 DPF for the indicated groupings (i.e., females with stable fracture), with all other nodes colored grey (i.e., those not in the indicated group). **(C)** For female (left) and male (right) mice, the degree of functional impairment is quantified as the distance of the post-fracture nodes to the baseline node (i.e., 5 DPI to baseline). Distance is measured in edges (weighted by the strength of the correlation between two nodes). Significant differences (p < 0.05 after corrections for multiple comparisons) are indicated by: § - unstable fracture and respective naive control; ‡ - stable fracture and respective naive control; * - unstable and stable fracture.

We next used a graph theory framework to analyze and visualize changes across our combined dataset. Graph theory allows for a more comprehensive global representation of all possible combinations of pairwise relationships in a single analysis and can identify patterns across the entire dataset, including behavioral state transitions associated with different datapoints within a group. In Figure 5A, each point on the graph is considered a “node” and represents an individual recording (i.e., one recording from 1 mouse at one timepoint). Connections between nodes are called “edges” and signify the strength of the correlation between data (i.e., quantified behavioral recordings) from two nodes (i.e., animals). Clusters of nodes connected by a high density of edges indicate a higher degree of correlation, or similarity, between those individual behavioral patterns. Groupings of nodes that are farther from each other, and that are connected by a lower density of edges, indicate less correlation. Using k-means clustering, we identified three separate clusters within our dataset (Figure 5A). The first two clusters correspond to non-fractured mice or late fracture timepoints (presumably when the animal has functionally recovered) for each sex (**cluster 1**: male, top left grouping of nodes vs. **cluster 2**: female, bottom left groupings of nodes). The third cluster is overwhelmingly represented by the early timepoints of the fractured mice (**cluster 3**: fracture, the right grouping of nodes). Importantly, as is shown in Figure 5B, nodes associated with individual animals shift their position within the graph over the time course of fracture related deficits and recovery. In early timepoints after the fracture, nodes are localized to the cluster defined by the fracture phenotype (cluster 3). However, as fracture healing occurs and mice functionally recover, nodes belonging to later timepoints progressively shift toward the naive groupings for each sex (**clusters 1 and 2**).

To assess functional recovery, we quantified differences in behavioral states between any given timepoint (e.g., 5-DPF) and the baseline, unfractured state (Figure 5C). Here, we quantified the distance between nodes, which in graph theoretic terms refers to the shortest path length, in number of edges, that the node must traverse to reach the baseline node from the corresponding animal. We interpret the distance to the baseline node as how close the animal is to achieving functional recovery. We found that for both female and male mice, the distance to baseline for the fracture condition for both stable and unstable fractures is significantly different from naïve mice at early timepoints post-fracture (5-10 DPF). However, at later timepoints (19-35 DPF), differences between fractured mice and naïve mice largely dissipated.

Lastly, we used mixed-effects modeling to compare the rate of return between fixation groups and sex on our unified gait metric. This comparison revealed a significant interaction between fixation x time (F (5, 26) = 7.009, p < 0.0003). No significant interactions were observed between sex x time (F (5, 26) = 0.9923, p < 0.44), sex x fixation (F (1, 26) = 0.07679, p < 0.78), or sex x fixation x time (F (5, 26) = 1.811, p < 0.15). These results further confirm that stable fractures recovered significantly faster than unstable fractures, and that sex did not influence recovery.

## Discussion

Although stabilized and unstabilized preclinical fracture models have been used frequently, no studies directly compared functional recovery between these models. The present study presents comprehensive behavioral phenotyping of the functional deficits and recovery time course of mice following fracture, when fracture fixation and sex are varied in a controlled manner. To accomplish this, we combined longitudinal behavioral imaging analysis, machine learning, and graph theory analysis to rapidly identify and quantify variables that could ultimately translate meaningfully to human changes in weightbearing and gait post-fracture. Consistent with clinical expectation, we found that tibia fractures with intramedullary stabilization present with less severe behavioral shifts compared to unstabilized fractures. On the other hand, we did not find that sex contributes to differences in functional healing when behavioral metrics were viewed together through our unified metric of recovery.

To date, there has been a critical gap in technology that can quickly and reliably longitudinally quantify functional recovery in rodent models following fracture. Existing tools for preclinical gait analysis include DigiGait and TreadScan, which use transparent treadmills to identify abnormalities in rodent walking patterns, or the CatWalk, which requires that animals are trained to walk along a narrow glass platform and then produces pressure maps of the mouse paws (Xu et al., 2019; Jacobs et al., 2014; Chen et al., 2017). The major limitation of these behavioral analyses is that the required training of each mouse is time consuming and also that the confined walking environment prevents animals from behaving naturally and ambulating freely. As our data clearly show, after a fracture, an animal’s balance and gait are compromised, limiting the effectiveness of forced gait tests which are highly dependent on stereotyped behaviors and parameters, including locomotor speed, consistent movement, and motivation (Pitzer et al., 2021). Data analysis using these methods is also laborious, making it challenging to perform high throughput screening. Here, we present the first use of the Blackbox (Zhang et al., 2022), a behavioral imaging system and illustrate how weightbearing and kinematic changes that occur during fracture injury and recovery can be readily monitored. This novel behavioral phenotyping approach rapidly identifies and quantifies fracture-related behavioral changes, overcoming many of the challenges associated with other systems.

To validate our behavioral phenotyping methodology, we chose to modulate clinically meaningful variables that, based on altered mechanobiology, are hypothesized to impact functional recovery following fracture. First, we changed the degree of fracture stabilization. There is substantial evidence that the interfragmentary motion at the fracture site is a major contributor to the extent of biological healing response. For long bone fractures, moderate motion and compressive forces promote the stem cell proliferation and differentiation required to support fracture healing. However, excessive motion (especially in sheer) or ridged fixation that eliminates mechanical loading can lead to non-union and chronic pain (Andrzejowski and Giannoudis, 2019; Hak et al., 2010; Tay et al., 2014; Brinker et al., 2013; Brinker et al., 2017). In clinical settings, this desired degree of mechanical loading for tibia fractures is most commonly achieved through surgical fixation with intramedullary nails. Intramedullary nails are believed to allow sufficient interfragmentary motion for optimal healing and enable patients to bear weight earlier (Gradl, 2014; Duan et al., 2012). We model this scenario in the mice with tibia fractures using intramedullary pin stabilization, which is well established in the field as a clinically relevant standard rodent model (Bonnarens and Einhorn, 1984). To model excessive motion we left the fractures unstabilized, which produces the same endochondral healing response, but with a high degree of interfragmentary motion (Le et al., 2001).

The post-fracture measurement time points were selected to ensure that behavior was recorded at each of the distinct phases of endochondral fracture healing (Marcucio et al., 2023; Bahney et al., 2019). For this reason, it was important to ensure that the post-operative analgesia provided by the sustained buprenorphine did not interfere with our analysis. Previous studies reported that sustained release buprenorphine can provide analgesia for up to 72 h in a fracture (Wolter et al., 2023) or hindpaw incision (Arthur et al., 2022) models, but less than 24 h in targeted inflammatory pain models (Larson et al., 2024). Thus, our first post-fracture recording at day 5, and by extension our extracted functional parameters, were not influenced by buprenorphine. Biologically, recordings at 5-DPF corresponds to the late hematoma phase, 7-DPF represents the soft/cartilage callus stage of healing, and 10-DPF captures a key timepoint during the conversion of cartilage to bone during endochondral ossification (Figures 1B,C). By 19-DPF, the callus has largely converted to trabeculated bone, with cortical bone remodeling occurring between 25- to 35-DPF.

We observed distinct functional recovery patterns in mice with pin-stabilized *versus* unstabilized fractures. The latter group demonstrated delayed weightbearing, take shorter steps, and their hind limb gait is more synchronous (normal gait is asynchronous). A limitation in the direct comparison of these two models is that our unstabilized fracture model also has more muscle damage adjacent to the fracture due to the three-point bending trauma. This trauma may have iatrogenic damage leading to fracture fragmentation when compared to the pin-stabilized model, which we make using a drill hole that permits better visualization of the pin insertion and ensures no fracture comminution. On the other hand, the pin-stabilized fracture can lead to some intraarticular injury during the pin insertion. Despite this limitation both models are common in the literature and correspond to clinically relevant fixation strategies (i.e., casting *versus* intramedullary nailing).

The clinical translatability of these findings is supported by human biomotion and loading studies that collectively demonstrate gait parameters and kinetic measures are reliable, objective indicators of functional recovery following lower extremity fractures (Agres et al., 2024; Alves et al., 2022a; Elsoe and Larsen, 2017; Larsen et al., 2017). Together these studies demonstrate weight bearing capacity, ground reaction forces, and gait symmetry directly reflect the restoration of underlying musculoskeletal function, with asymmetric patterns persisting up to 1 year, indicating incomplete recovery. Although weightbearing metrics do not directly translate to internal forces at the fracture site (Heyland et al., 2023), the predictable relationship between kinetic forces and internal joint loading, combined with progressive normalization of gait parameters over time, does establish gait analysis as both a diagnostic tool and quantitative outcome measure for assessing functional recovery. Our partial paw intensity metrics (i.e., percent weightbearing on toes, pad, heal), which considers the weight distribution across different parts of the hindpaw, also has complementary clinical support as proxy for functional fracture healing (Heyland et al., 2023; Falzarano et al., 2018).

Collectively, existing data on sex differences in fracture recovery are inconclusive as to whether there are clinically meaningful alterations in functional recovery or pain behavior (Ortona et al., 2023). Consistent with other studies (Pitzer et al., 2021; Broom et al., 2021), we first show that unfractured, age-matched female and male mice exhibit significantly different gait and kinematic parameters, likely due in part to the heavier gross weights of male mice relative to females. Consequently, all post-fracture behavioral data in our study were normalized to naïve mice of the same sex. After this adjustment, we did not detect major sex-specific differences in functional recovery patterns, supporting our earlier histomorophometric data showing equivalent bone healing between male and female mice response after the difference in animal weight was taken into account (Wong et al., 2020a). Consistent with our finding, Tawfik et al. (2020) did not find sex-specific differences in fracture responses using either von Frey fibers or gait analysis. On the other hand, as the Tawfik et al and other studies have shown sex-specific divergence in innate and adaptive immune cell response after fracture, with a stronger immune response to injury in females, the observed lack of behavioral differences between sexes was somewhat surprising a (Tawfik et al., 2020; Molitoris et al., 2024b) Clinical data have found sex differences in acute pain that are dependent upon the location of the fracture (Tighe et al., 2014) and in a non-fracture setting there is evidence that females have higher incidence of pain, are more sensitive to painful stimulation as assessed in the laboratory, and are more likely to develop chronic pain (45% incidence *versus* 31%) (Rollman and Lautenbacher, 2001; Fillingim et al., 2009; Mogil and Bailey, 2010; Tsang et al., 2008).

Interpreting high dimensional datasets is an ongoing challenge, particularly when analyzing complex behavioral changes in response to injury. To address this challenge, we introduced a novel graph theoretic framework to analyze our combined datasets. This approach enables a global representation of large, complex datasets into a single framework and facilitates estimation of simple and interpretable summary metrics. Our unified metric, the distance to the baseline node in graph theoretic space, made it possible to identify when fractured mice can reasonably be considered to have “recovered”. To the best of our knowledge, this is the first application of graph theory to analyze state changes in kinematic animal behaviors.

Despite differences in individual metrics at later fracture timepoints, our unified metric establishes that composite functional deficits only persist until 10 DPF, with both male and female mice largely recovered by 19 DPF. This functional timeline coincides with the shift from cartilage to bone in the fracture callus (Bahney et al., 2019). The biological timeline of healing is well established in the fracture literature and it is known that at 5 DPF there is a robust pro-inflammatory response and a large hematoma within the fracture gap; as expected all mice at 5 DPF presented with severe functional deficits. Functional recovery begins between 7 and 10 DPF, correlating with the formation and maturation of cartilage within the fracture callus. By 19 DPF there is substantial trabecular bone formation bridging the fracture and at this time point we found that there are no longer significant functional deficits in any of the mice. Other studies did report that gait-related changes persist (for 4–6 weeks) in mice with a femur fracture, however, these heal more slowly than tibia fractures, and these studies only reported the traditional single parameter view of functional recovery (Hofman et al., 2020; Magnusdottir et al., 2021).

This study is a critical first step to addressing a technology gap in quantifying functional recovery following fracture in a preclinical model system. With our behavioral phenotyping approach we can rapidly and sensitively capture, quantify, and interpret a broad array of weightbearing and kinematic parameters. Furthermore, our unified graph theoretic metric of fracture recovery is flexible and can easily be repurposed for other studies. Importantly, this metric was highly sensitive to system variations (surgical fixation and sex), even with only 5 animals per group, and enabled non-destructive longitudinal analysis. In future studies, we plan to integrate our behavioral phenotyping approach with biological and/or mechanical healing parameters of the fracture. We also aim to expand our behavioral phenotyping approach to improve screening of therapeutics that can accelerate functional bone healing and analgesics that treat fracture pain.

As to translational significance, our preclinical behavioral phenotyping approach correlates with biomotion and loading studies conducted in the clinic although functional recovery is likely dependent upon both fracture location and activity (Alves et al., 2022b). Behavioral phenotyping in the clinic could also benefit from including patient reported outcomes (PROs) related to pain or physical function scores, such as the lower extremity function score (LEFS, (Binkley et al., 1999)) in our case of tibia fracture. Importantly, we believe that the introduction of graph theory as a methodology can integrate multiple variables into a single outcome measure of functional fracture healing. Graph theory could immediately synthesize clinical data sets that include standard of care outcomes, such as radiographic scoring (mRUST), with PROs or with emerging techniques that to capture function (biomotion, loading, FIX-IT (Bhandari et al., 2013)) or biological status (blood based biomarkers to immune function or bone healing).

## Data Availability

The raw data supporting the conclusions of this article will be made available by the authors, without undue reservation.

## Supplementary material

SUPPLEMENTARY FIGURE S1 Deeplabcut labeling strategy. Crosses correspond to points used for labeling for training pose estimation models within Deeplabcut.

SUPPLEMENTARY FIGURE S2 Sex differences in kinematic endpoints in naïve mice at baseline. Graphs illustrate sex differences in **(A)** body weight (g; Student’s t-test, p < 0.0001); **(B)** paw FTIR intensity (AU; Student’s t-test, p = 0.0092); **(C)** step duration (ms; Student’s t-test, p < 0.0001); **(D)** step length (cm; Student’s t-test, p < 0.0001); and **(E)** maximum paw speed (cm/s; Student’s t-test, p < 0.0001).

SUPPLEMENTARY FIGURE S3 Paw FTIR intensity correlates with mouse weight. Paw FTIR intensity significantly correlates with mouse weight during **(A)** stepping (Y = 2065*X+21041; p = 0.0147; *R* ^2^ = 0.2082) and while **(B)** at rest, not stepping (Y = 3,730*X+398.3; p = 0.0048; *R* ^2^ = 0.2769). Solid line indicates best fit line after simple linear regression, and dotted lines indicate 95% confidence intervals.

SUPPLEMENTARY FIGURE S4 Z-scored heat map of all behavioral metrics over time. The x-axis of heatmap are represented by the different behavioral metrics assessed. The y-axis corresponds to data for a single condition (time, sex, fracture) averaged across all animals within that particular condition (M = male; F = female; BL = baseline; No-SFx or No-UFx indicate naïve controls; SFx and UFx indicate stabilized and unstablized fractures). In both dimensions, data are organized after ordering by hierarchical clustering analysis. Associated dendrograms are appended to each axis.

SUPPLEMENTARY FIGURE S5 Pairwise correlation matrix by behaviors over time. The x- and y-axis correspond to pairwise correlations across behavioral metrics between individual time points. Individual pixels are colored by correlation strength (Pearson’s correlation). Legend: (M = male; F = female; BL = baseline; No-SFx or No-UFx indicate naïve controls; SFx and UFx indicate stabilized and unstablized fractures). In both dimensions, data are organized after ordering by hierarchical clustering analysis, employing the same organization as the y-axis in Supplementary Figure S4

SUPPLEMENTARY FIGURE S6 Principal component analysis of functional changes after tibia fracture. **(A)** Functional changes after tibia fracture are plotted using principal component analysis. Individual recordings are represented as smaller circles in the graph, and group means are represented as larger circles. The representation of group identities on the graph, as indicated by sex and fracture conditions. Colored circles indicated data from Baseline to 35 DPF for the indicated groupings (i.e., females with stable fracture), with all other circles colored grey (i.e., those not in the indicated group). **(B)** Loading coefficient weighting for principal components 1 and 2, organized using hierarchical clustering analysis.
