# Supplementary material for: Deep behavioral phenotyping tracks functional recovery following tibia fracture in mice
Source: Front Physiol. 2025 Aug 26;16:1630155. doi: 10.3389/fphys.2025.1630155 (PMC12417503; doi:10.3389/fphys.2025.1630155)
Supplement: Supplementary file 2 [file Supplementaryfile1.docx]

|  |  |  |  |  |
| --- | --- | --- | --- | --- |
| **FIGURE 2B STATISTICS** |  |  |  |  |
| Table Analyzed | Female_Weightbearing Ratio Log2 (Fractured/Intact) | | |  |
|  |  |  |  |  |
| Mixed-effects model (REML) | Matching by factors: Row factor & (AB vs CD) | |  |  |
| Assume sphericity? | Yes |  |  |  |
| Alpha | 0.05 |  |  |  |
|  |  |  |  |  |
| Fixed effects (type III) | P value | P value summary | Statistically significant (P < 0.05)? | F (DFn, DFd) |
| Row factor | <0.0001 | **** | Yes | F (6, 48) = 30.89 |
| (AB vs CD) | <0.0001 | **** | Yes | F (1, 8) = 80.68 |
| (AC vs BD) | 0.5362 | ns | No | F (1, 8) = 0.4176 |
| Row factor x (AB vs CD) | <0.0001 | **** | Yes | F (6, 48) = 36.59 |
| Row factor x (AC vs BD) | 0.0308 | * | Yes | F (6, 48) = 2.567 |
| (AB vs CD) x (AC vs BD) | 0.0459 | * | Yes | F (1, 8) = 5.570 |
| Row factor x (AB vs CD) x (AC vs BD) | 0.0002 | *** | Yes | F (6, 48) = 5.448 |
|  |  |  |  |  |
| Random effects | SD | Variance |  |  |
| Subject | 0 | 0 |  |  |
| Subject x Row factor | 0.0313 | 0.0009799 |  |  |
| Subject x (AB vs CD) | 0.08458 | 0.007154 |  |  |
| Residual | 0.1131 | 0.01278 |  |  |
|  |  |  |  |  |
| Was the matching effective? |  |  |  |  |
| Chi-square, df | 21.68, 2 |  |  |  |
| P value | <0.0001 |  |  |  |
| P value summary | **** |  |  |  |
| Is there significant matching (P < 0.05)? | Yes |  |  |  |
|  |  |  |  |  |
| Data summary |  |  |  |  |
| Number of columns | 2 x 2 |  |  |  |
| Number of rows (Row factor) | 7 |  |  |  |
| Number of subjects (Subject) | 10 |  |  |  |
| Number of missing values | 0 |  |  |  |

**FIGURE 2C STATISTICS**

| Table Analyzed | Female_Pad Intensity (%Total Paw) | | |  |
| --- | --- | --- | --- | --- |
|  |  |  |  |  |
| Mixed-effects model (REML) | Matching by factors: Row factor & (AB vs CD) | |  |  |
| Assume sphericity? | Yes |  |  |  |
| Alpha | 0.05 |  |  |  |
|  |  |  |  |  |
| Fixed effects (type III) | P value | P value summary | Statistically significant (P < 0.05)? | F (DFn, DFd) |
| Row factor | <0.0001 | **** | Yes | F (6, 48) = 25.07 |
| (AB vs CD) | 0.0011 | ** | Yes | F (1, 8) = 24.65 |
| (AC vs BD) | 0.0334 | * | Yes | F (1, 8) = 6.579 |
| Row factor x (AB vs CD) | <0.0001 | **** | Yes | F (6, 48) = 27.62 |
| Row factor x (AC vs BD) | <0.0001 | **** | Yes | F (6, 48) = 8.547 |
| (AB vs CD) x (AC vs BD) | 0.005 | ** | Yes | F (1, 8) = 14.64 |
| Row factor x (AB vs CD) x (AC vs BD) | <0.0001 | **** | Yes | F (6, 48) = 6.178 |
|  |  |  |  |  |
| Random effects | SD | Variance |  |  |
| Subject | 1.168 | 1.364 |  |  |
| Subject x Row factor | 0 | 0 |  |  |
| Subject x (AB vs CD) | 2.699 | 7.286 |  |  |
| Residual | 3.837 | 14.72 |  |  |
|  |  |  |  |  |
| Was the matching effective? |  |  |  |  |
| Chi-square, df | 25.78, 2 |  |  |  |
| P value | <0.0001 |  |  |  |
| P value summary | **** |  |  |  |
| Is there significant matching (P < 0.05)? | Yes |  |  |  |
|  |  |  |  |  |
| Data summary |  |  |  |  |
| Number of columns | 2 x 2 |  |  |  |
| Number of rows (Row factor) | 7 |  |  |  |
| Number of subjects (Subject) | 10 |  |  |  |
| Number of missing values | 0 |  |  |  |

**FIGURE 2D STATISTICS**

| Table Analyzed | Female_Stepping Cprrelation (Pearson's Rho) | |  |  |
| --- | --- | --- | --- | --- |
|  |  |  |  |  |
| Mixed-effects model (REML) | Matching by factors: Row factor & (AB vs CD) | |  |  |
| Assume sphericity? | Yes |  |  |  |
| Alpha | 0.05 |  |  |  |
|  |  |  |  |  |
| Fixed effects (type III) | P value | P value summary | Statistically significant (P < 0.05)? | F (DFn, DFd) |
| Row factor | <0.0001 | **** | Yes | F (6, 48) = 14.13 |
| (AB vs CD) | 0.0291 | * | Yes | F (1, 8) = 7.037 |
| (AC vs BD) | 0.0271 | * | Yes | F (1, 8) = 7.281 |
| Row factor x (AB vs CD) | <0.0001 | **** | Yes | F (6, 48) = 12.79 |
| Row factor x (AC vs BD) | <0.0001 | **** | Yes | F (6, 48) = 6.436 |
| (AB vs CD) x (AC vs BD) | 0.0139 | * | Yes | F (1, 8) = 9.839 |
| Row factor x (AB vs CD) x (AC vs BD) | <0.0001 | **** | Yes | F (6, 48) = 7.739 |
|  |  |  |  |  |
| Random effects | SD | Variance |  |  |
| Subject | 0 | 0 |  |  |
| Subject x Row factor | 0 | 0 |  |  |
| Subject x (AB vs CD) | 0.02052 | 0.0004209 |  |  |
| Residual | 0.06346 | 0.004027 |  |  |
|  |  |  |  |  |
| Was the matching effective? |  |  |  |  |
| Chi-square, df | 2.349, 1 |  |  |  |
| P value | 0.1254 |  |  |  |
| P value summary | ns |  |  |  |
| Is there significant matching (P < 0.05)? | No |  |  |  |
|  |  |  |  |  |
| Data summary |  |  |  |  |
| Number of columns | 2 x 2 |  |  |  |
| Number of rows (Row factor) | 7 |  |  |  |
| Number of subjects (Subject) | 10 |  |  |  |
| Number of missing values | 0 |  |  |  |

**FIGURE 2E STATISTICS**

| Table Analyzed | Female_Step Duration (Right Hindpaw; ms) | |  |  |
| --- | --- | --- | --- | --- |
|  |  |  |  |  |
| Mixed-effects model (REML) | Matching by factors: Row factor & (AB vs CD) | |  |  |
| Assume sphericity? | Yes |  |  |  |
| Alpha | 0.05 |  |  |  |
|  |  |  |  |  |
| Fixed effects (type III) | P value | P value summary | Statistically significant (P < 0.05)? | F (DFn, DFd) |
| Row factor | <0.0001 | **** | Yes | F (6, 48) = 17.39 |
| (AB vs CD) | <0.0001 | **** | Yes | F (1, 8) = 113.2 |
| (AC vs BD) | 0.6369 | ns | No | F (1, 8) = 0.2407 |
| Row factor x (AB vs CD) | <0.0001 | **** | Yes | F (6, 48) = 20.00 |
| Row factor x (AC vs BD) | 0.154 | ns | No | F (6, 48) = 1.650 |
| (AB vs CD) x (AC vs BD) | 0.5849 | ns | No | F (1, 8) = 0.3239 |
| Row factor x (AB vs CD) x (AC vs BD) | 0.3559 | ns | No | F (6, 48) = 1.136 |
|  |  |  |  |  |
| Random effects | SD | Variance |  |  |
| Subject | 1.332 | 1.774 |  |  |
| Subject x Row factor | 0.8648 | 0.7479 |  |  |
| Subject x (AB vs CD) | 0.7742 | 0.5994 |  |  |
| Residual | 3.459 | 11.97 |  |  |
|  |  |  |  |  |
| Was the matching effective? |  |  |  |  |
| Chi-square, df | 7.761, 3 |  |  |  |
| P value | 0.0512 |  |  |  |
| P value summary | ns |  |  |  |
| Is there significant matching (P < 0.05)? | No |  |  |  |
|  |  |  |  |  |
| Data summary |  |  |  |  |
| Number of columns | 2 x 2 |  |  |  |
| Number of rows (Row factor) | 7 |  |  |  |
| Number of subjects (Subject) | 10 |  |  |  |
| Number of missing values | 0 |  |  |  |

**FIGURE 2F STATISTICS**

| Table Analyzed | Female_Maxpawspeed (Right, Hindpaw, cm/s) | |  |  |
| --- | --- | --- | --- | --- |
|  |  |  |  |  |
| Mixed-effects model (REML) | Matching by factors: Row factor & (AB vs CD) | |  |  |
| Assume sphericity? | Yes |  |  |  |
| Alpha | 0.05 |  |  |  |
|  |  |  |  |  |
| Fixed effects (type III) | P value | P value summary | Statistically significant (P < 0.05)? | F (DFn, DFd) |
| Row factor | <0.0001 | **** | Yes | F (6, 48) = 9.228 |
| (AB vs CD) | 0.017 | * | Yes | F (1, 8) = 9.027 |
| (AC vs BD) | 0.0758 | ns | No | F (1, 8) = 4.158 |
| Row factor x (AB vs CD) | <0.0001 | **** | Yes | F (6, 48) = 15.72 |
| Row factor x (AC vs BD) | 0.0322 | * | Yes | F (6, 48) = 2.543 |
| (AB vs CD) x (AC vs BD) | 0.6177 | ns | No | F (1, 8) = 0.2696 |
| Row factor x (AB vs CD) x (AC vs BD) | 0.1706 | ns | No | F (6, 48) = 1.590 |
|  |  |  |  |  |
| Random effects | SD | Variance |  |  |
| Subject | 0 | 0 |  |  |
| Subject x Row factor | 0.9494 | 0.9015 |  |  |
| Subject x (AB vs CD) | 2.65 | 7.021 |  |  |
| Residual | 2.482 | 6.161 |  |  |
|  |  |  |  |  |
| Was the matching effective? |  |  |  |  |
| Chi-square, df | 43.84, 2 |  |  |  |
| P value | <0.0001 |  |  |  |
| P value summary | **** |  |  |  |
| Is there significant matching (P < 0.05)? | Yes |  |  |  |
|  |  |  |  |  |
| Data summary |  |  |  |  |
| Number of columns | 2 x 2 |  |  |  |
| Number of rows (Row factor) | 7 |  |  |  |
| Number of subjects (Subject) | 10 |  |  |  |
| Number of missing values | 0 |  |  |  |

**FIGURE 2G STATISTICS**

| Table Analyzed | Female_Step Length (Right Hindpaw; cm) | |  |  |
| --- | --- | --- | --- | --- |
|  |  |  |  |  |
| Mixed-effects model (REML) | Matching by factors: Row factor & (AB vs CD) | |  |  |
| Assume sphericity? | Yes |  |  |  |
| Alpha | 0.05 |  |  |  |
|  |  |  |  |  |
| Fixed effects (type III) | P value | P value summary | Statistically significant (P < 0.05)? | F (DFn, DFd) |
| Row factor | 0.4648 | ns | No | F (6, 48) = 0.9560 |
| (AB vs CD) | 0.9134 | ns | No | F (1, 8) = 0.01261 |
| (AC vs BD) | 0.1516 | ns | No | F (1, 8) = 2.512 |
| Row factor x (AB vs CD) | 0.2084 | ns | No | F (6, 48) = 1.470 |
| Row factor x (AC vs BD) | 0.0999 | ns | No | F (6, 48) = 1.901 |
| (AB vs CD) x (AC vs BD) | 0.0652 | ns | No | F (1, 8) = 4.561 |
| Row factor x (AB vs CD) x (AC vs BD) | 0.5335 | ns | No | F (6, 48) = 0.8564 |
|  |  |  |  |  |
| Random effects | SD | Variance |  |  |
| Subject | 0 | 0 |  |  |
| Subject x Row factor | 0.04132 | 0.001708 |  |  |
| Subject x (AB vs CD) | 0.1103 | 0.01217 |  |  |
| Residual | 0.1573 | 0.02475 |  |  |
|  |  |  |  |  |
| Was the matching effective? |  |  |  |  |
| Chi-square, df | 19.09, 2 |  |  |  |
| P value | <0.0001 |  |  |  |
| P value summary | **** |  |  |  |
| Is there significant matching (P < 0.05)? | Yes |  |  |  |
|  |  |  |  |  |
| Data summary |  |  |  |  |
| Number of columns | 2 x 2 |  |  |  |
| Number of rows (Row factor) | 7 |  |  |  |
| Number of subjects (Subject) | 10 |  |  |  |
| Number of missing values | 0 |  |  |  |

**FIGURE 3B STATISTICS**

| Table Analyzed | Male_Weightbearing Ratio Log2 (Fractured/Intact) | | |  |
| --- | --- | --- | --- | --- |
|  |  |  |  |  |
| Mixed-effects model (REML) | Matching by factors: Row factor & (AB vs CD) | |  |  |
| Assume sphericity? | Yes |  |  |  |
| Alpha | 0.05 |  |  |  |
|  |  |  |  |  |
| Fixed effects (type III) | P value | P value summary | Statistically significant (P < 0.05)? | F (DFn, DFd) |
| Row factor | <0.0001 | **** | Yes | F (6, 42) = 10.79 |
| (AB vs CD) | 0.0003 | *** | Yes | F (1, 7) = 42.48 |
| (AC vs BD) | 0.0352 | * | Yes | F (1, 7) = 6.786 |
| Row factor x (AB vs CD) | <0.0001 | **** | Yes | F (6, 41) = 10.12 |
| Row factor x (AC vs BD) | 0.6597 | ns | No | F (6, 42) = 0.6888 |
| (AB vs CD) x (AC vs BD) | 0.0891 | ns | No | F (1, 7) = 3.892 |
| Row factor x (AB vs CD) x (AC vs BD) | 0.6305 | ns | No | F (6, 41) = 0.7269 |
|  |  |  |  |  |
| Random effects | SD | Variance |  |  |
| Subject | 0.06967 | 0.004854 |  |  |
| Subject x Row factor | 0.02975 | 0.0008853 |  |  |
| Subject x (AB vs CD) | 0.06315 | 0.003988 |  |  |
| Residual | 0.2498 | 0.06241 |  |  |
|  |  |  |  |  |
| Was the matching effective? |  |  |  |  |
| Chi-square, df | 3.828, 3 |  |  |  |
| P value | 0.2806 |  |  |  |
| P value summary | ns |  |  |  |
| Is there significant matching (P < 0.05)? | No |  |  |  |
|  |  |  |  |  |
| Data summary |  |  |  |  |
| Number of columns | 2 x 2 |  |  |  |
| Number of rows (Row factor) | 7 |  |  |  |
| Number of subjects (Subject) | 9 |  |  |  |
| Number of missing values | 1 |  |  |  |

**FIGURE 3C STATISTICS**

| Table Analyzed | Male_Pad Intensity (% Total Paw) | |  |  |
| --- | --- | --- | --- | --- |
|  |  |  |  |  |
| Mixed-effects model (REML) | Matching by factors: Row factor & (AB vs CD) | |  |  |
| Assume sphericity? | Yes |  |  |  |
| Alpha | 0.05 |  |  |  |
|  |  |  |  |  |
| Fixed effects (type III) | P value | P value summary | Statistically significant (P < 0.05)? | F (DFn, DFd) |
| Row factor | <0.0001 | **** | Yes | F (6, 42) = 24.90 |
| (AB vs CD) | <0.0001 | **** | Yes | F (1, 7) = 64.59 |
| (AC vs BD) | 0.0118 | * | Yes | F (1, 7) = 11.40 |
| Row factor x (AB vs CD) | <0.0001 | **** | Yes | F (6, 42) = 25.78 |
| Row factor x (AC vs BD) | <0.0001 | **** | Yes | F (6, 42) = 11.55 |
| (AB vs CD) x (AC vs BD) | 0.0055 | ** | Yes | F (1, 7) = 15.65 |
| Row factor x (AB vs CD) x (AC vs BD) | <0.0001 | **** | Yes | F (6, 42) = 9.506 |
|  |  |  |  |  |
| Random effects | SD | Variance |  |  |
| Subject | 0 | 0 |  |  |
| Subject x Row factor | 0 | 0 |  |  |
| Subject x (AB vs CD) | 1.168 | 1.364 |  |  |
| Residual | 3.419 | 11.69 |  |  |
|  |  |  |  |  |
| Was the matching effective? |  |  |  |  |
| Chi-square, df | 2.456, 1 |  |  |  |
| P value | 0.117 |  |  |  |
| P value summary | ns |  |  |  |
| Is there significant matching (P < 0.05)? | No |  |  |  |
|  |  |  |  |  |
| Data summary |  |  |  |  |
| Number of columns | 2 x 2 |  |  |  |
| Number of rows (Row factor) | 7 |  |  |  |
| Number of subjects (Subject) | 9 |  |  |  |
| Number of missing values | 0 |  |  |  |

**FIGURE 3D STATISTICS**

| Table Analyzed | Male_Stepping Correlation (Pearson's Rho) | |  |  |
| --- | --- | --- | --- | --- |
|  |  |  |  |  |
| Mixed-effects model (REML) | Matching by factors: Row factor & (AB vs CD) | |  |  |
| Assume sphericity? | Yes |  |  |  |
| Alpha | 0.05 |  |  |  |
|  |  |  |  |  |
| Fixed effects (type III) | P value | P value summary | Statistically significant (P < 0.05)? | F (DFn, DFd) |
| Row factor | <0.0001 | **** | Yes | F (6, 42) = 6.387 |
| (AB vs CD) | 0.0121 | * | Yes | F (1, 7) = 11.30 |
| (AC vs BD) | 0.4907 | ns | No | F (1, 7) = 0.5287 |
| Row factor x (AB vs CD) | 0.0283 | * | Yes | F (6, 42) = 2.656 |
| Row factor x (AC vs BD) | 0.0009 | *** | Yes | F (6, 42) = 4.749 |
| (AB vs CD) x (AC vs BD) | 0.0937 | ns | No | F (1, 7) = 3.760 |
| Row factor x (AB vs CD) x (AC vs BD) | 0.0352 | * | Yes | F (6, 42) = 2.528 |
|  |  |  |  |  |
| Random effects | SD | Variance |  |  |
| Subject | 0 | 0 |  |  |
| Subject x Row factor | 0 | 0 |  |  |
| Subject x (AB vs CD) | 0.02121 | 0.0004498 |  |  |
| Residual | 0.07119 | 0.005068 |  |  |
|  |  |  |  |  |
| Was the matching effective? |  |  |  |  |
| Chi-square, df | 1.568, 1 |  |  |  |
| P value | 0.2105 |  |  |  |
| P value summary | ns |  |  |  |
| Is there significant matching (P < 0.05)? | No |  |  |  |
|  |  |  |  |  |
| Data summary |  |  |  |  |
| Number of columns | 2 x 2 |  |  |  |
| Number of rows (Row factor) | 7 |  |  |  |
| Number of subjects (Subject) | 9 |  |  |  |
| Number of missing values | 0 |  |  |  |

**FIGURE 3E STATISTICS**

| Table Analyzed | Male_Step Duration (Right Hindpaw; ms) | |  |  |
| --- | --- | --- | --- | --- |
|  |  |  |  |  |
| Mixed-effects model (REML) | Matching by factors: Row factor & (AB vs CD) | |  |  |
| Assume sphericity? | No |  |  |  |
| Alpha | 0.05 |  |  |  |
|  |  |  |  |  |
| Fixed effects (type III) | P value | P value summary | Statistically significant (P < 0.05)? | F (DFn, DFd) |
| Row factor | <0.0001 | **** | Yes | F (6.000, 42.00) = 33.92 |
| (AB vs CD) | 0.0059 | ** | Yes | F (0.4867, 3.407) = 45.07 |
| (AC vs BD) | 0.0939 | ns | No | F (1, 7) = 3.754 |
| Row factor x (AB vs CD) | <0.0001 | **** | Yes | F (2.661, 18.63) = 34.09 |
| Row factor x (AC vs BD) | 0.6021 | ns | No | F (6, 42) = 0.7643 |
| (AB vs CD) x (AC vs BD) | 0.2034 | ns | No | F (1, 7) = 1.968 |
| Row factor x (AB vs CD) x (AC vs BD) | 0.4488 | ns | No | F (6, 42) = 0.9832 |
|  |  |  |  |  |
| Random effects | SD | Variance |  |  |
| Subject | 0 | 0 |  |  |
| Subject x Row factor | 0 | 0 |  |  |
| Subject x (AB vs CD) | 1.569 | 2.461 |  |  |
| Residual | 2.101 | 4.416 |  |  |
|  |  |  |  |  |
| Was the matching effective? |  |  |  |  |
| Chi-square, df | 22.99, 1 |  |  |  |
| P value | <0.0001 |  |  |  |
| P value summary | **** |  |  |  |
| Is there significant matching (P < 0.05)? | Yes |  |  |  |
|  |  |  |  |  |
| Data summary |  |  |  |  |
| Number of columns | 2 x 2 |  |  |  |
| Number of rows (Row factor) | 7 |  |  |  |
| Number of subjects (Subject) | 9 |  |  |  |
| Number of missing values | 0 |  |  |  |

**FIGURE 3F STATISTICS**

| Table Analyzed | Male_Maximum Speed (Right Hindpaw, cm/s) | |  |  |
| --- | --- | --- | --- | --- |
|  |  |  |  |  |
| Mixed-effects model (REML) | Matching by factors: Row factor & (AB vs CD) | |  |  |
| Assume sphericity? | Yes |  |  |  |
| Alpha | 0.05 |  |  |  |
|  |  |  |  |  |
| Fixed effects (type III) | P value | P value summary | Statistically significant (P < 0.05)? | F (DFn, DFd) |
| Row factor | 0.0004 | *** | Yes | F (6, 42) = 5.237 |
| (AB vs CD) | 0.1325 | ns | No | F (1, 7) = 2.898 |
| (AC vs BD) | 0.6517 | ns | No | F (1, 7) = 0.2222 |
| Row factor x (AB vs CD) | 0.0013 | ** | Yes | F (6, 42) = 4.526 |
| Row factor x (AC vs BD) | 0.0006 | *** | Yes | F (6, 42) = 5.003 |
| (AB vs CD) x (AC vs BD) | 0.2553 | ns | No | F (1, 7) = 1.535 |
| Row factor x (AB vs CD) x (AC vs BD) | 0.4517 | ns | No | F (6, 42) = 0.9786 |
|  |  |  |  |  |
| Random effects | SD | Variance |  |  |
| Subject | 0 | 0 |  |  |
| Subject x Row factor | 0 | 0 |  |  |
| Subject x (AB vs CD) | 1.639 | 2.687 |  |  |
| Residual | 2.118 | 4.486 |  |  |
|  |  |  |  |  |
| Was the matching effective? |  |  |  |  |
| Chi-square, df | 22.94, 1 |  |  |  |
| P value | <0.0001 |  |  |  |
| P value summary | **** |  |  |  |
| Is there significant matching (P < 0.05)? | Yes |  |  |  |
|  |  |  |  |  |
| Data summary |  |  |  |  |
| Number of columns | 2 x 2 |  |  |  |
| Number of rows (Row factor) | 7 |  |  |  |
| Number of subjects (Subject) | 9 |  |  |  |
| Number of missing values | 0 |  |  |  |

**FIGURE 3E STATISTICS**

| Table Analyzed | Male_Step Length (Right Hindpaw, cm) | |  |  |
| --- | --- | --- | --- | --- |
|  |  |  |  |  |
| Mixed-effects model (REML) | Matching by factors: Row factor & (AB vs CD) | |  |  |
| Assume sphericity? | Yes |  |  |  |
| Alpha | 0.05 |  |  |  |
|  |  |  |  |  |
| Fixed effects (type III) | P value | P value summary | Statistically significant (P < 0.05)? | F (DFn, DFd) |
| Row factor | 0.0003 | *** | Yes | F (6, 42) = 5.508 |
| (AB vs CD) | 0.5203 | ns | No | F (1, 7) = 0.4581 |
| (AC vs BD) | 0.7606 | ns | No | F (1, 7) = 0.1004 |
| Row factor x (AB vs CD) | 0.029 | * | Yes | F (6, 42) = 2.642 |
| Row factor x (AC vs BD) | 0.1947 | ns | No | F (6, 42) = 1.522 |
| (AB vs CD) x (AC vs BD) | 0.5013 | ns | No | F (1, 7) = 0.5026 |
| Row factor x (AB vs CD) x (AC vs BD) | 0.6906 | ns | No | F (6, 42) = 0.6490 |
|  |  |  |  |  |
| Random effects | SD | Variance |  |  |
| Subject | 0 | 0 |  |  |
| Subject x Row factor | 0 | 0 |  |  |
| Subject x (AB vs CD) | 0.1322 | 0.01747 |  |  |
| Residual | 0.1595 | 0.02544 |  |  |
|  |  |  |  |  |
| Was the matching effective? |  |  |  |  |
| Chi-square, df | 26.61, 1 |  |  |  |
| P value | <0.0001 |  |  |  |
| P value summary | **** |  |  |  |
| Is there significant matching (P < 0.05)? | Yes |  |  |  |
|  |  |  |  |  |
| Data summary |  |  |  |  |
| Number of columns | 2 x 2 |  |  |  |
| Number of rows (Row factor) | 7 |  |  |  |
| Number of subjects (Subject) | 9 |  |  |  |
| Number of missing values | 0 |  |  |  |

**FIGURE 4A STATISTICS**

| Table Analyzed | Male and Female_Weightbearing Ratio Log2 (Fractured/Intact) | | |  |
| --- | --- | --- | --- | --- |
|  |  |  |  |  |
| Mixed-effects model (REML) | Matching by factors: Row factor & (AB vs CD) | |  |  |
| Assume sphericity? | Yes |  |  |  |
| Alpha | 0.05 |  |  |  |
|  |  |  |  |  |
| Fixed effects (type III) | P value | P value summary | Statistically significant (P < 0.05)? | F (DFn, DFd) |
| Row factor | <0.0001 | **** | Yes | F (6, 48) = 41.07 |
| (AB vs CD) | 0.258 | ns | No | F (1, 8) = 1.483 |
| (AC vs BD) | 0.0087 | ** | Yes | F (1, 8) = 11.92 |
| Row factor x (AB vs CD) | 0.0609 | ns | No | F (6, 41) = 2.214 |
| Row factor x (AC vs BD) | 0.0352 | * | Yes | F (6, 48) = 2.493 |
| (AB vs CD) x (AC vs BD) | 0.2112 | ns | No | F (1, 8) = 1.847 |
| Row factor x (AB vs CD) x (AC vs BD) | 0.6734 | ns | No | F (6, 41) = 0.6711 |
|  |  |  |  |  |
| Random effects | SD | Variance |  |  |
| Subject | 0 | 0 |  |  |
| Subject x Row factor | 0 | 0 |  |  |
| Subject x (AB vs CD) | 0.1007 | 0.01015 |  |  |
| Residual | 0.2275 | 0.05176 |  |  |
|  |  |  |  |  |
| Was the matching effective? |  |  |  |  |
| Chi-square, df | 5.838, 1 |  |  |  |
| P value | 0.0157 |  |  |  |
| P value summary | * |  |  |  |
| Is there significant matching (P < 0.05)? | Yes |  |  |  |
|  |  |  |  |  |
| Data summary |  |  |  |  |
| Number of columns | 2 x 2 |  |  |  |
| Number of rows (Row factor) | 7 |  |  |  |
| Number of subjects (Subject) | 10 |  |  |  |
| Number of missing values | 7 |  |  |  |

**FIGURE 4B STATISTICS**

| Table Analyzed | Male and Female_Pad Intensity (% Total Paw) | |  |  |
| --- | --- | --- | --- | --- |
|  |  |  |  |  |
| Mixed-effects model (REML) | Matching by factors: Row factor & (AB vs CD) | |  |  |
| Assume sphericity? | Yes |  |  |  |
| Alpha | 0.05 |  |  |  |
|  |  |  |  |  |
| Fixed effects (type III) | P value | P value summary | Statistically significant (P < 0.05)? | F (DFn, DFd) |
| Row factor | <0.0001 | **** | Yes | F (6, 48) = 60.35 |
| (AB vs CD) | 0.1258 | ns | No | F (1, 8) = 2.922 |
| (AC vs BD) | 0.0035 | ** | Yes | F (1, 8) = 16.65 |
| Row factor x (AB vs CD) | 0.312 | ns | No | F (6, 41) = 1.228 |
| Row factor x (AC vs BD) | <0.0001 | **** | Yes | F (6, 48) = 20.60 |
| (AB vs CD) x (AC vs BD) | 0.2754 | ns | No | F (1, 8) = 1.370 |
| Row factor x (AB vs CD) x (AC vs BD) | 0.5106 | ns | No | F (6, 41) = 0.8908 |
|  |  |  |  |  |
| Random effects | SD | Variance |  |  |
| Subject | 2.264 | 5.124 |  |  |
| Subject x Row factor | 1.846 | 3.409 |  |  |
| Subject x (AB vs CD) | 2.116 | 4.477 |  |  |
| Residual | 3.852 | 14.83 |  |  |
|  |  |  |  |  |
| Was the matching effective? |  |  |  |  |
| Chi-square, df | 24.36, 3 |  |  |  |
| P value | <0.0001 |  |  |  |
| P value summary | **** |  |  |  |
| Is there significant matching (P < 0.05)? | Yes |  |  |  |
|  |  |  |  |  |
| Data summary |  |  |  |  |
| Number of columns | 2 x 2 |  |  |  |
| Number of rows (Row factor) | 7 |  |  |  |
| Number of subjects (Subject) | 10 |  |  |  |
| Number of missing values | 7 |  |  |  |

**FIGURE 4C STATISTICS**

| Table Analyzed | Male and Female_Steppinf Correlation (Pearson's Rho) | | |  |
| --- | --- | --- | --- | --- |
|  |  |  |  |  |
| Mixed-effects model (REML) | Matching by factors: Row factor & (AB vs CD) | |  |  |
| Assume sphericity? | Yes |  |  |  |
| Alpha | 0.05 |  |  |  |
|  |  |  |  |  |
| Fixed effects (type III) | P value | P value summary | Statistically significant (P < 0.05)? | F (DFn, DFd) |
| Row factor | <0.0001 | **** | Yes | F (6, 48) = 26.38 |
| (AB vs CD) | 0.7143 | ns | No | F (1, 8) = 0.1439 |
| (AC vs BD) | 0.0055 | ** | Yes | F (1, 8) = 14.19 |
| Row factor x (AB vs CD) | 0.1696 | ns | No | F (6, 41) = 1.608 |
| Row factor x (AC vs BD) | <0.0001 | **** | Yes | F (6, 48) = 16.47 |
| (AB vs CD) x (AC vs BD) | 0.2591 | ns | No | F (1, 8) = 1.476 |
| Row factor x (AB vs CD) x (AC vs BD) | 0.7196 | ns | No | F (6, 41) = 0.6116 |
|  |  |  |  |  |
| Random effects | SD | Variance |  |  |
| Subject | 0 | 0 |  |  |
| Subject x Row factor | 0.02247 | 0.0005048 |  |  |
| Subject x (AB vs CD) | 0.02425 | 0.0005882 |  |  |
| Residual | 0.06439 | 0.004146 |  |  |
|  |  |  |  |  |
| Was the matching effective? |  |  |  |  |
| Chi-square, df | 3.156, 2 |  |  |  |
| P value | 0.2064 |  |  |  |
| P value summary | ns |  |  |  |
| Is there significant matching (P < 0.05)? | No |  |  |  |
|  |  |  |  |  |
| Data summary |  |  |  |  |
| Number of columns | 2 x 2 |  |  |  |
| Number of rows (Row factor) | 7 |  |  |  |
| Number of subjects (Subject) | 10 |  |  |  |
| Number of missing values | 7 |  |  |  |

**FIGURE 4D STATISTICS**

| Table Analyzed | Male and Female_Normalized Step Duration (Right Hindpaw) | | |  |
| --- | --- | --- | --- | --- |
|  |  |  |  |  |
| Mixed-effects model (REML) | Matching by factors: Row factor & (AB vs CD) | |  |  |
| Assume sphericity? | Yes |  |  |  |
| Alpha | 0.05 |  |  |  |
|  |  |  |  |  |
| Fixed effects (type III) | P value | P value summary | Statistically significant (P < 0.05)? | F (DFn, DFd) |
| Row factor | <0.0001 | **** | Yes | F (6, 48) = 44.44 |
| (AB vs CD) | 0.4803 | ns | No | F (1, 8) = 0.5480 |
| (AC vs BD) | 0.2054 | ns | No | F (1, 8) = 1.900 |
| Row factor x (AB vs CD) | 0.4682 | ns | No | F (6, 41) = 0.9536 |
| Row factor x (AC vs BD) | 0.4507 | ns | No | F (6, 48) = 0.9776 |
| (AB vs CD) x (AC vs BD) | 0.5321 | ns | No | F (1, 8) = 0.4263 |
| Row factor x (AB vs CD) x (AC vs BD) | 0.4773 | ns | No | F (6, 41) = 0.9398 |
|  |  |  |  |  |
| Random effects | SD | Variance |  |  |
| Subject | 0 | 0 |  |  |
| Subject x Row factor | 0 | 0 |  |  |
| Subject x (AB vs CD) | 0.06789 | 0.004608 |  |  |
| Residual | 0.1254 | 0.01573 |  |  |
|  |  |  |  |  |
| Was the matching effective? |  |  |  |  |
| Chi-square, df | 10.25, 1 |  |  |  |
| P value | 0.0014 |  |  |  |
| P value summary | ** |  |  |  |
| Is there significant matching (P < 0.05)? | Yes |  |  |  |
|  |  |  |  |  |
| Data summary |  |  |  |  |
| Number of columns | 2 x 2 |  |  |  |
| Number of rows (Row factor) | 7 |  |  |  |
| Number of subjects (Subject) | 10 |  |  |  |
| Number of missing values | 7 |  |  |  |

**FIGURE 4E STATISTICS**

| Table Analyzed | Male and Female_Normalized Maximum Speed (Right Hindpaw) | | |  |
| --- | --- | --- | --- | --- |
|  |  |  |  |  |
| Mixed-effects model (REML) | Matching by factors: Row factor & (AB vs CD) | |  |  |
| Assume sphericity? | Yes |  |  |  |
| Alpha | 0.05 |  |  |  |
|  |  |  |  |  |
| Fixed effects (type III) | P value | P value summary | Statistically significant (P < 0.05)? | F (DFn, DFd) |
| Row factor | <0.0001 | **** | Yes | F (6, 48) = 15.30 |
| (AB vs CD) | 0.3819 | ns | No | F (1, 8) = 0.8561 |
| (AC vs BD) | 0.8493 | ns | No | F (1, 8) = 0.03853 |
| Row factor x (AB vs CD) | 0.4304 | ns | No | F (6, 41) = 1.013 |
| Row factor x (AC vs BD) | 0.3984 | ns | No | F (6, 48) = 1.062 |
| (AB vs CD) x (AC vs BD) | 0.27 | ns | No | F (1, 8) = 1.405 |
| Row factor x (AB vs CD) x (AC vs BD) | 0.2378 | ns | No | F (6, 41) = 1.400 |
|  |  |  |  |  |
| Random effects | SD | Variance |  |  |
| Subject | 0 | 0 |  |  |
| Subject x Row factor | 0 | 0 |  |  |
| Subject x (AB vs CD) | 0.1242 | 0.01543 |  |  |
| Residual | 0.1125 | 0.01266 |  |  |
|  |  |  |  |  |
| Was the matching effective? |  |  |  |  |
| Chi-square, df | 49.86, 1 |  |  |  |
| P value | <0.0001 |  |  |  |
| P value summary | **** |  |  |  |
| Is there significant matching (P < 0.05)? | Yes |  |  |  |
|  |  |  |  |  |
| Data summary |  |  |  |  |
| Number of columns | 2 x 2 |  |  |  |
| Number of rows (Row factor) | 7 |  |  |  |
| Number of subjects (Subject) | 10 |  |  |  |
| Number of missing values | 7 |  |  |  |

**FIGURE 4F STATISTICS**

| Table Analyzed | Male and Female_Normalized Step Length (Right Hindpaw) | | |  |
| --- | --- | --- | --- | --- |
|  |  |  |  |  |
| Mixed-effects model (REML) | Matching by factors: Row factor & (AB vs CD) | |  |  |
| Assume sphericity? | Yes |  |  |  |
| Alpha | 0.05 |  |  |  |
|  |  |  |  |  |
| Fixed effects (type III) | P value | P value summary | Statistically significant (P < 0.05)? | F (DFn, DFd) |
| Row factor | 0.6278 | ns | No | F (6, 48) = 0.7299 |
| (AB vs CD) | 0.5225 | ns | No | F (1, 8) = 0.4472 |
| (AC vs BD) | 0.9927 | ns | No | F (1, 8) = 8.901e-005 |
| Row factor x (AB vs CD) | 0.0636 | ns | No | F (6, 41) = 2.189 |
| Row factor x (AC vs BD) | 0.7646 | ns | No | F (6, 48) = 0.5538 |
| (AB vs CD) x (AC vs BD) | 0.195 | ns | No | F (1, 8) = 2.000 |
| Row factor x (AB vs CD) x (AC vs BD) | 0.751 | ns | No | F (6, 41) = 0.5709 |
|  |  |  |  |  |
| Random effects | SD | Variance |  |  |
| Subject | 0 | 0 |  |  |
| Subject x Row factor | 0 | 0 |  |  |
| Subject x (AB vs CD) | 0.2152 | 0.04632 |  |  |
| Residual | 0.2263 | 0.0512 |  |  |
|  |  |  |  |  |
| Was the matching effective? |  |  |  |  |
| Chi-square, df | 37.77, 1 |  |  |  |
| P value | <0.0001 |  |  |  |
| P value summary | **** |  |  |  |
| Is there significant matching (P < 0.05)? | Yes |  |  |  |
|  |  |  |  |  |
| Data summary |  |  |  |  |
| Number of columns | 2 x 2 |  |  |  |
| Number of rows (Row factor) | 7 |  |  |  |
| Number of subjects (Subject) | 10 |  |  |  |
| Number of missing values | 7 |  |  |  |

| **FIGURE 5C STATISTICS**  **(Combined Model for Left and Right panel)** |  |  |  |  |  |
| --- | --- | --- | --- | --- | --- |
| Table Analyzed | PAW_GraphTheoryAnalysis-NodeDistance-Weighted |  |  |  |  |
|  |  |  |  |  |  |
| Mixed-effects model (REML) | Matching: Stacked |  |  |  |  |
| Assume sphericity? | Yes |  |  |  |  |
| Alpha | 0.05 |  |  |  |  |
|  |  |  |  |  |  |
| **Fixed effects (type III)** | **P value** | **P value**  **summary** | **Statistically**  **significant (P < 0.05)?** | **F (DFn, DFd)** |  |
| Row Factor | <0.0001 | **** | Yes | F (5, 137) = 9.604 |  |
| Column Factor | <0.0001 | **** | Yes | F (7, 28) = 7.634 |  |
| Row Factor x Column Factor | <0.0001 | **** | Yes | F (35, 137) = 4.696 |  |
|  |  |  |  |  |  |
| **Random effects** | **SD** | **Variance** |  |  |  |
| Subject | 0.1093 | 0.01194 |  |  |  |
| Residual | 0.1685 | 0.02839 |  |  |  |
|  |  |  |  |  |  |
| Was the matching effective? |  |  |  |  |  |
| Chi-square, df | 21.87, 1 |  |  |  |  |
| P value | <0.0001 |  |  |  |  |
| P value summary | **** |  |  |  |  |
| Is there significant matching (P < 0.05)? | Yes |  |  |  |  |
|  |  |  |  |  |  |
| Data summary |  |  |  |  |  |
| Number of columns (Column Factor) | 8 |  |  |  |  |
| Number of rows (Row Factor) | 6 |  |  |  |  |
| Number of subjects (Subject) | 36 |  |  |  |  |
| Number of missing values | 3 |  |  |  |  |
