# Supplementary material for: Deep behavioral phenotyping tracks functional recovery following tibia fracture in mice
Source: Front Physiol. 2025 Aug 26;16:1630155. doi: 10.3389/fphys.2025.1630155 (PMC12417503; doi:10.3389/fphys.2025.1630155)
Supplement: Supplementary file 3 [file DataSheet1.pdf]

Supplemental Figures for:

Deep behavioral phenotyping tracks functional recovery following tibia fracture in mice.

Layne et al, 2025. Frontiers in Physiology.

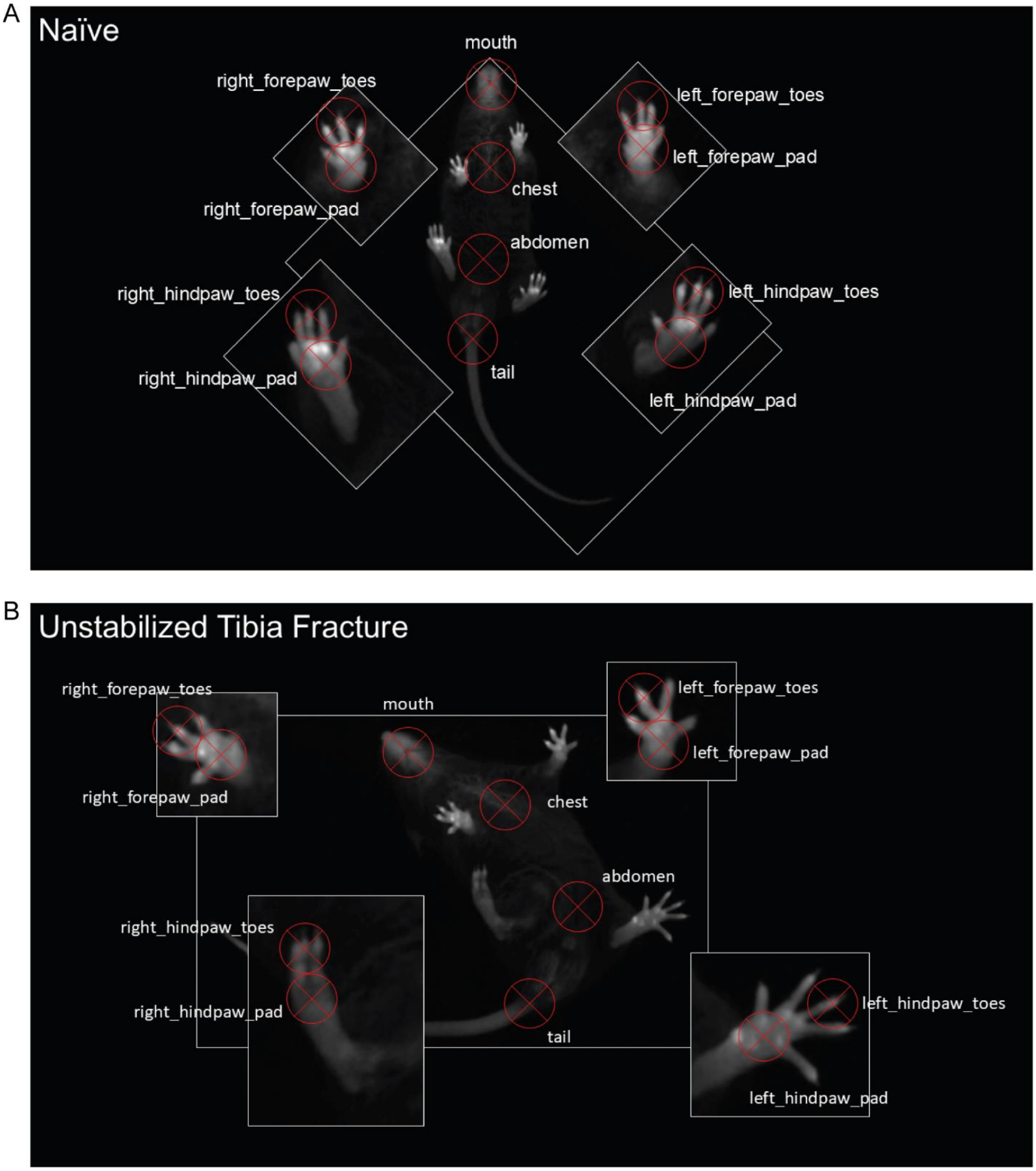

SUPPLEMENTARY FIGURE S1. Deeplabcut labeling strategy. Crosses correspond to points used for labeling for training pose estimation models within Deeplabcut.

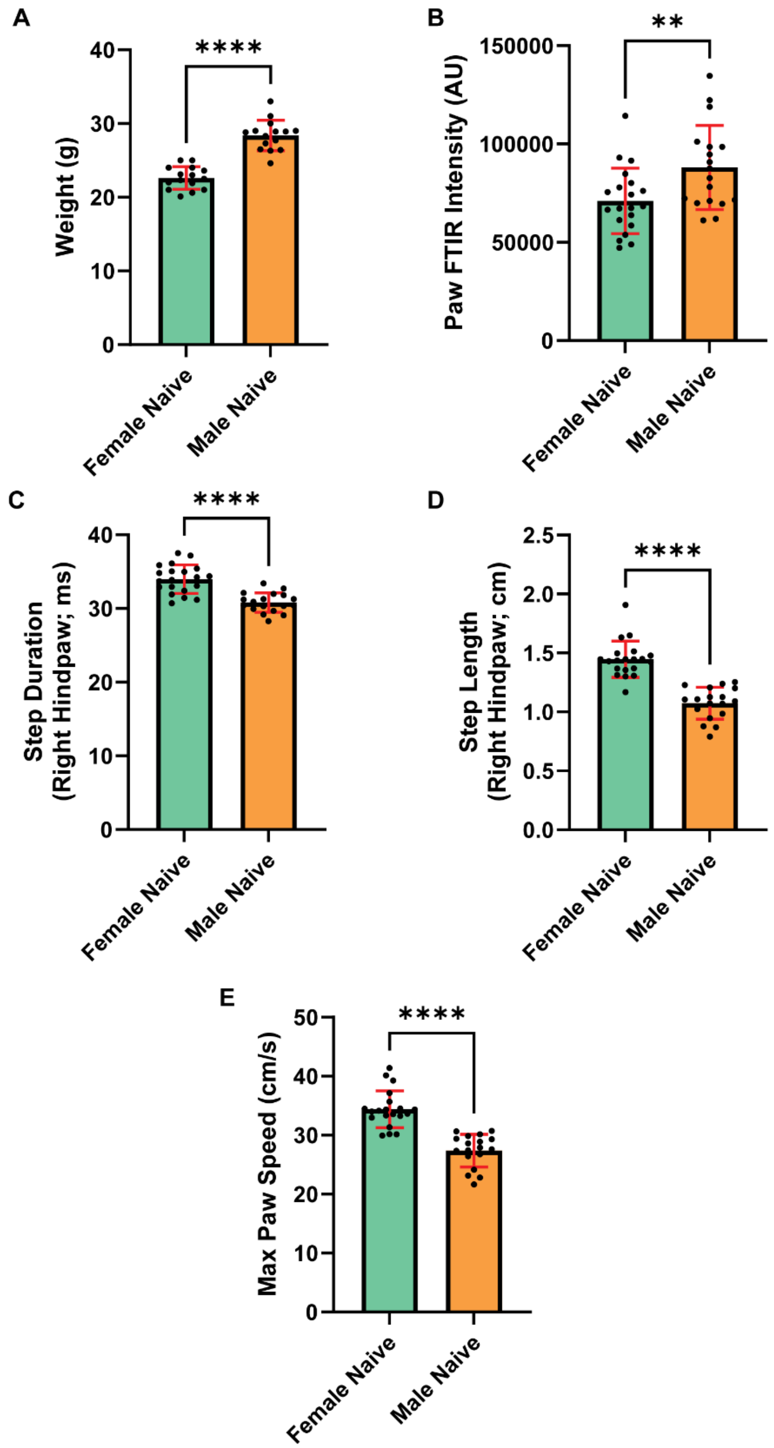

SUPPLEMENTARY FIGURE S2. Sex differences in kinematic endpoints in naïve mice at baseline. Graphs illustrate sex differences in (A) body weight (g; Student's t-test, $p < 0.0001$); (B) paw FTIR intensity (AU; Student's t-test, $p = 0.0092$); (C) step duration (ms; Student's t-test, $p < 0.0001$); (D) step length (cm; Student's t-test, $p < 0.0001$); and (E) maximum paw speed (cm/s; Student's t-test, $p < 0.0001$).

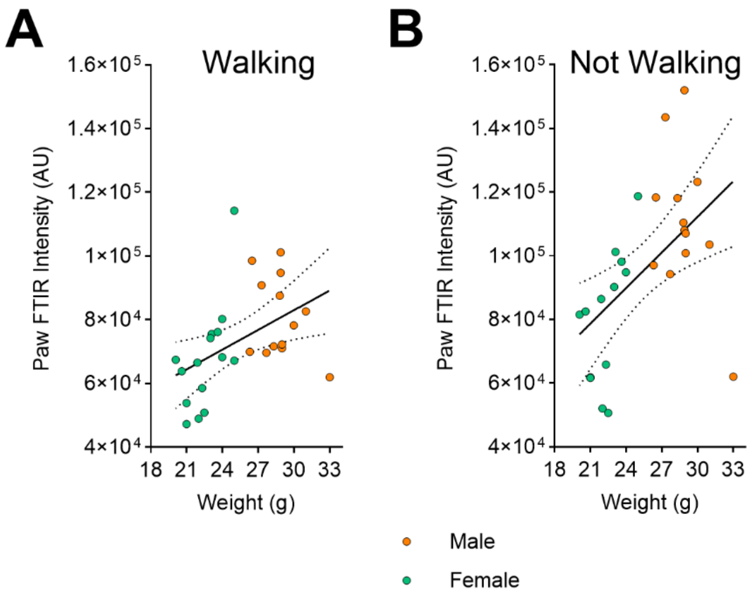

SUPPLEMENTARY FIGURE S3. Paw FTIR intensity correlates with mouse weight. Paw FTIR intensity significantly correlates with mouse weight during (A) stepping (Y = 2065∗X+21041; p = 0.0147; R2 = 0.2082) and while (B) at rest, not stepping (Y = 3,730∗X+398.3; p = 0.0048; R2 = 0.2769). Solid line indicates best fit line after simple linear regression, and dotted lines indicate 95% confidence intervals.

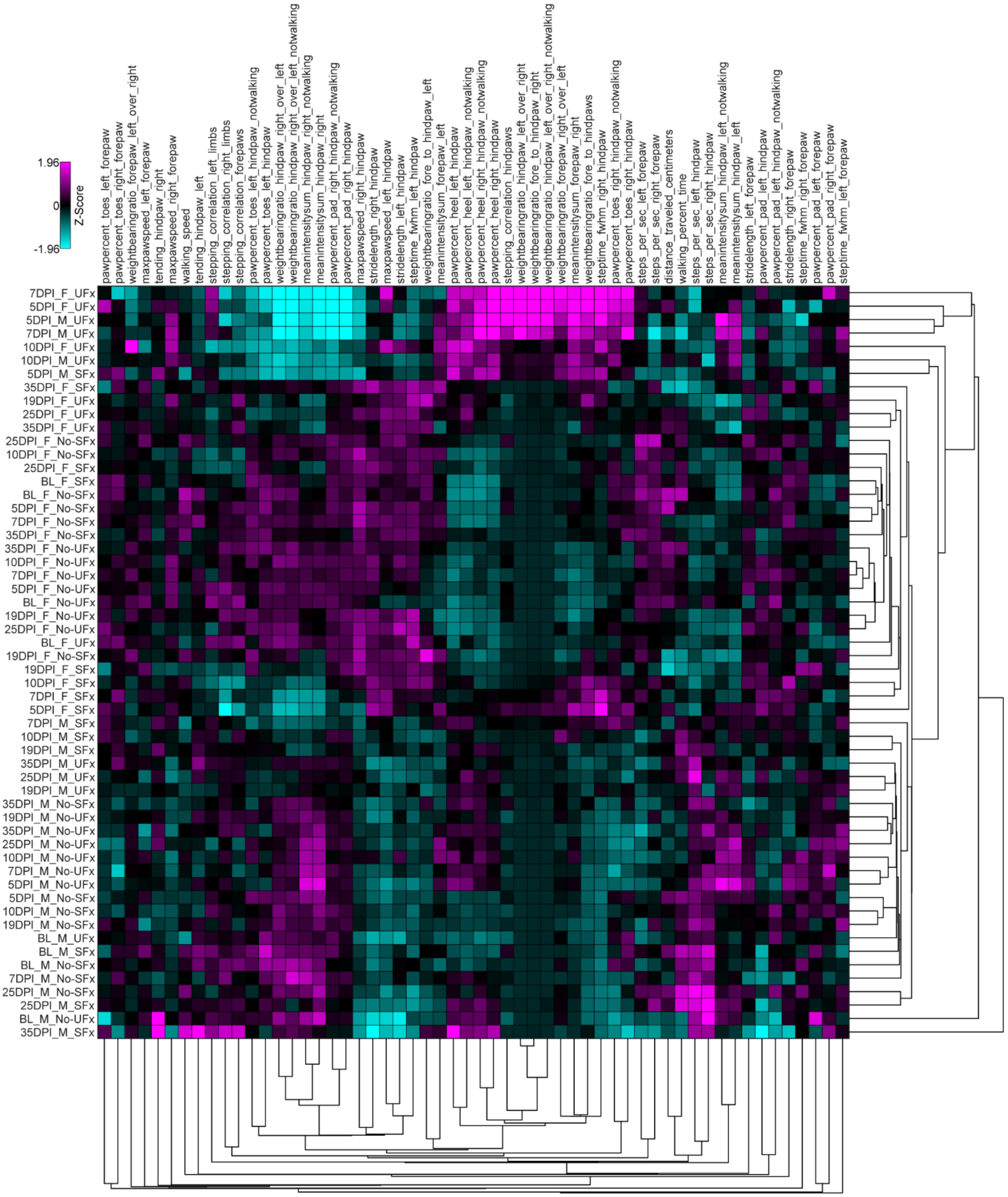

SUPPLEMENTARY FIGURE S4. Z-scored heat map of all behavioral metrics over time. The x-axis of heatmap are represented by the different behavioral metrics assessed. The y-axis corresponds to data for a single condition (time, sex, fracture) averaged across all animals within that particular condition (M = male; F = female; BL = baseline; No-SFx or No-UFx indicate naïve controls; SFx and UFx indicate stabilized and unstablized fractures). In both dimensions, data are organized after ordering by hierarchical clustering analysis. Associated dendrograms are appended to each axis.

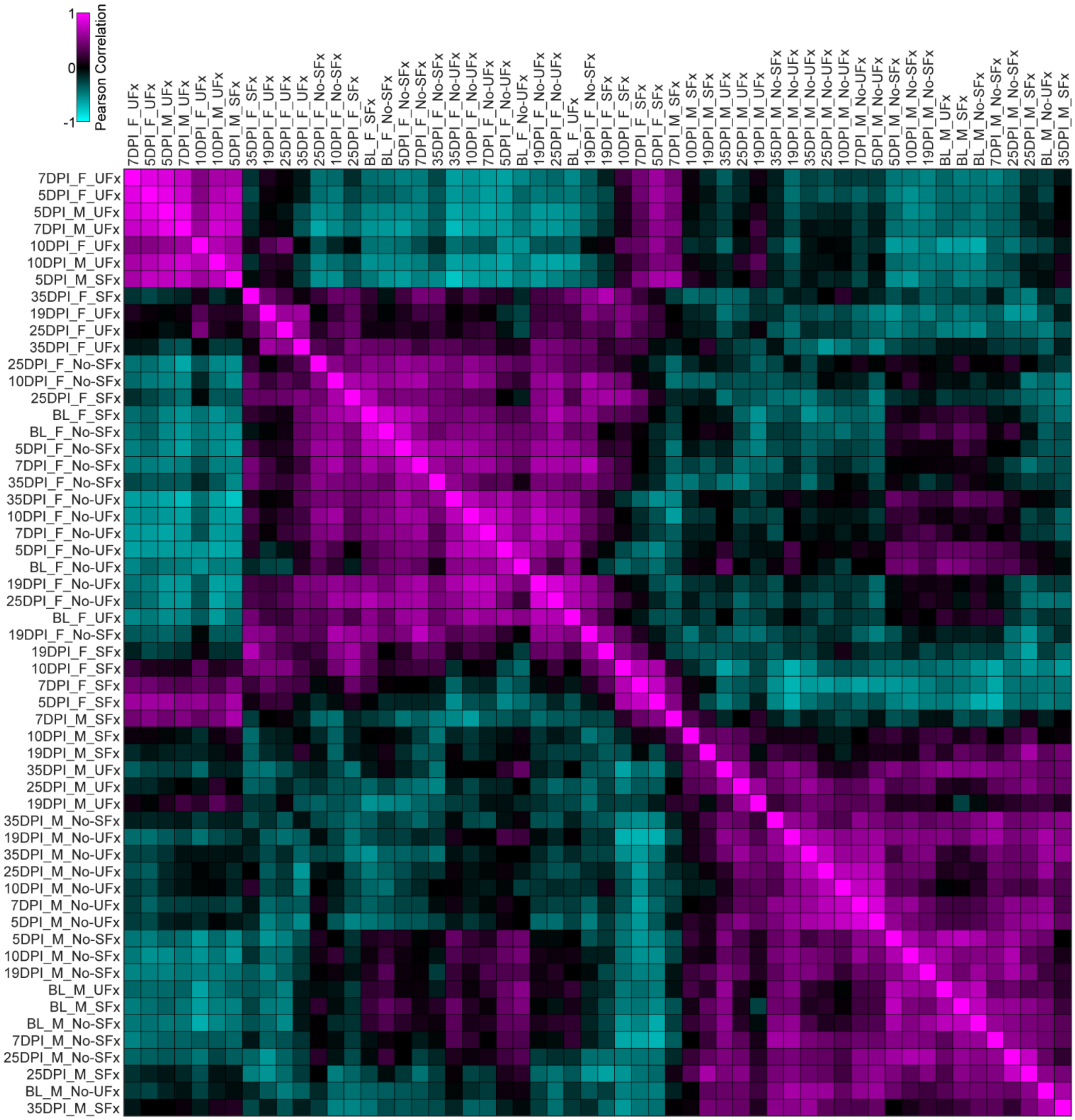

SUPPLEMENTARY FIGURE S5. Pairwise correlation matrix by behaviors over time. The x- and y-axis correspond to pairwise correlations across behavioral metrics between individual time points. Individual pixels are colored by correlation strength (Pearson's correlation). Legend: (M = male; F = female; BL = baseline; No-SFx or No-UFx indicate naïve controls; SFx and UFx indicate stabilized and unstablized fractures). In both dimensions, data are organized after ordering by hierarchical clustering analysis, employing the same organization as the y-axis in Supplementary Figure S4

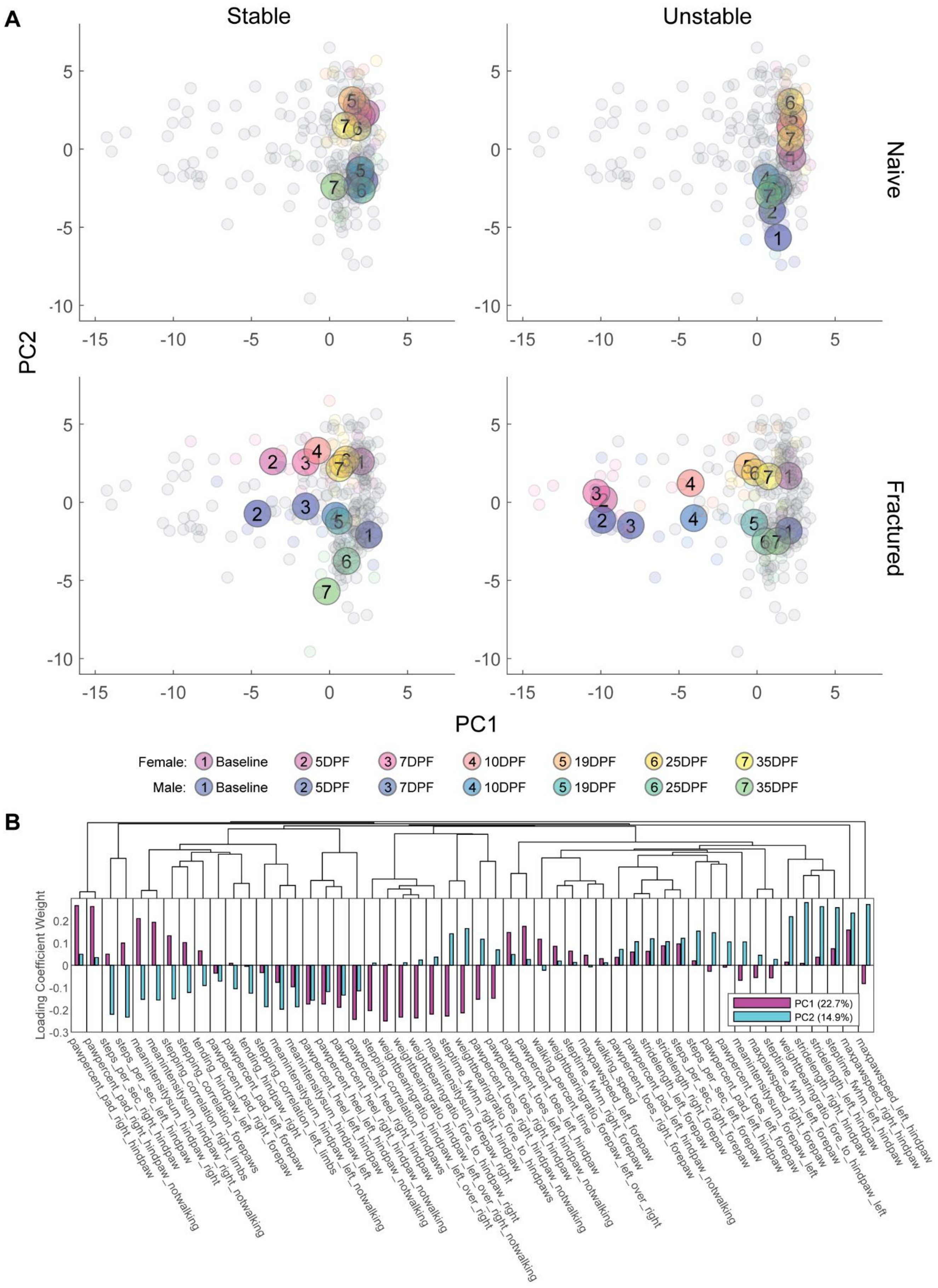

SUPPLEMENTARY FIGURE S6 (Continued on next page)

SUPPLEMENTARY FIGURE S6 (Continued from previous page)

Principal component analysis of functional changes after tibia fracture. (A) Functional changes after tibia fracture are plotted using principal component analysis. Individual recordings are represented as smaller circles in the graph, and group means are represented as larger circles. The representation of group identities on the graph, as indicated by sex and fracture conditions. Colored circles indicated data from Baseline to 35 DPF for the indicated groupings (i.e., females with stable fracture), with all other circles colored grey (i.e., those not in the indicated group). (B) Loading coefficient weighting for principal components 1 and 2, organized using hierarchical clustering analysis.
